# Supplementary figures and images for: A Large-Scale Conformational Change Couples Membrane Recruitment to Cargo Binding in the AP2 Clathrin Adaptor Complex
Source: Cell. 2010 Jun 25;141(7):1220–9. doi: 10.1016/j.cell.2010.05.006 (PMC3655264; doi:10.1016/j.cell.2010.05.006)

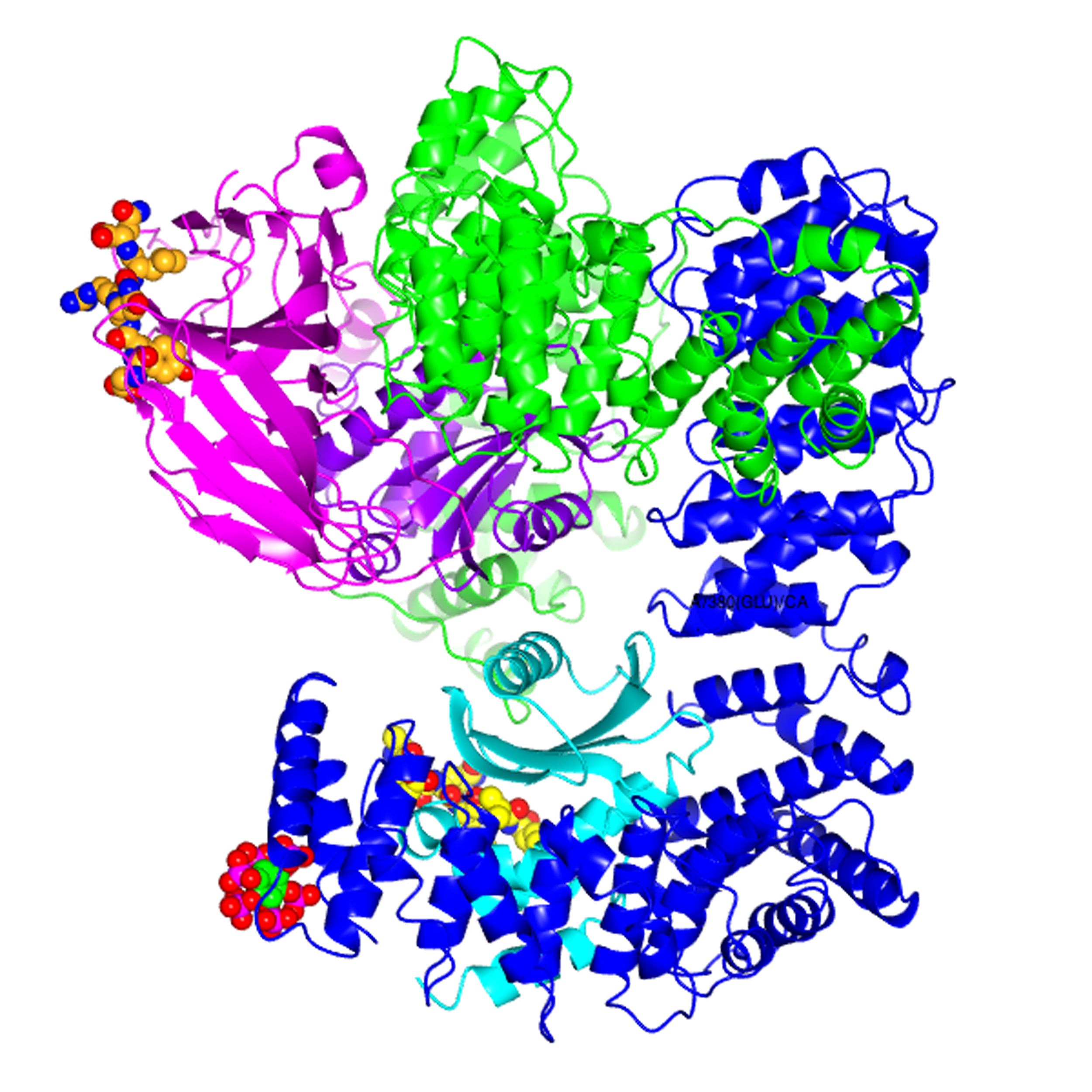

Supplement: Movie S1. Related to Figure 3 — Alternation of the locked and open conformations of AP2, in ribbon representation, showing the C-μ2 domain (magenta) moving out of the α-β2 bowl, exposing the motif-binding sites (spheres). [file mmc1.jpg]

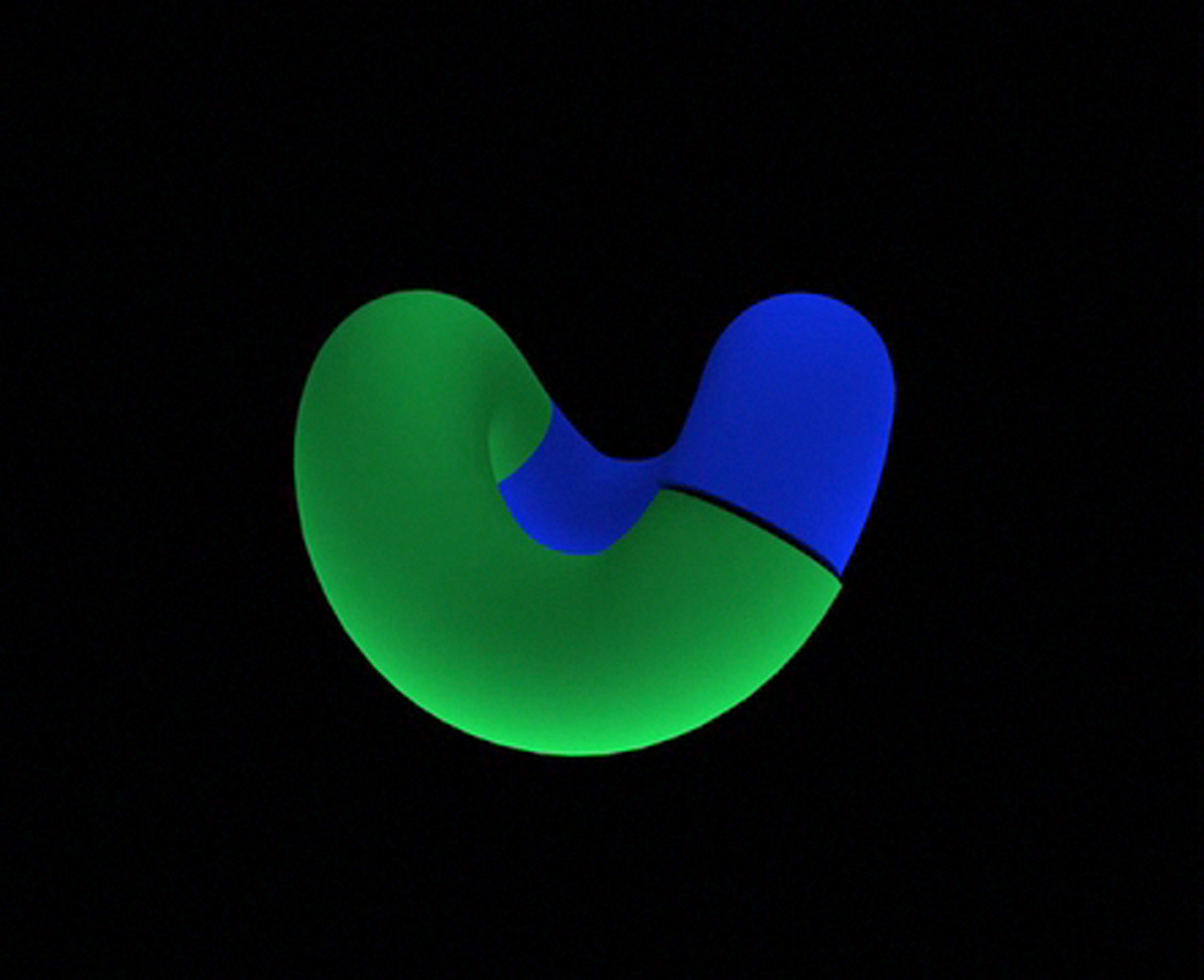

Supplement: Movie S2. Related to Figure 3 — Schematic representation of the conformational change in the α-β2 bowl (blue and green, respectively). [file mmc2.jpg]

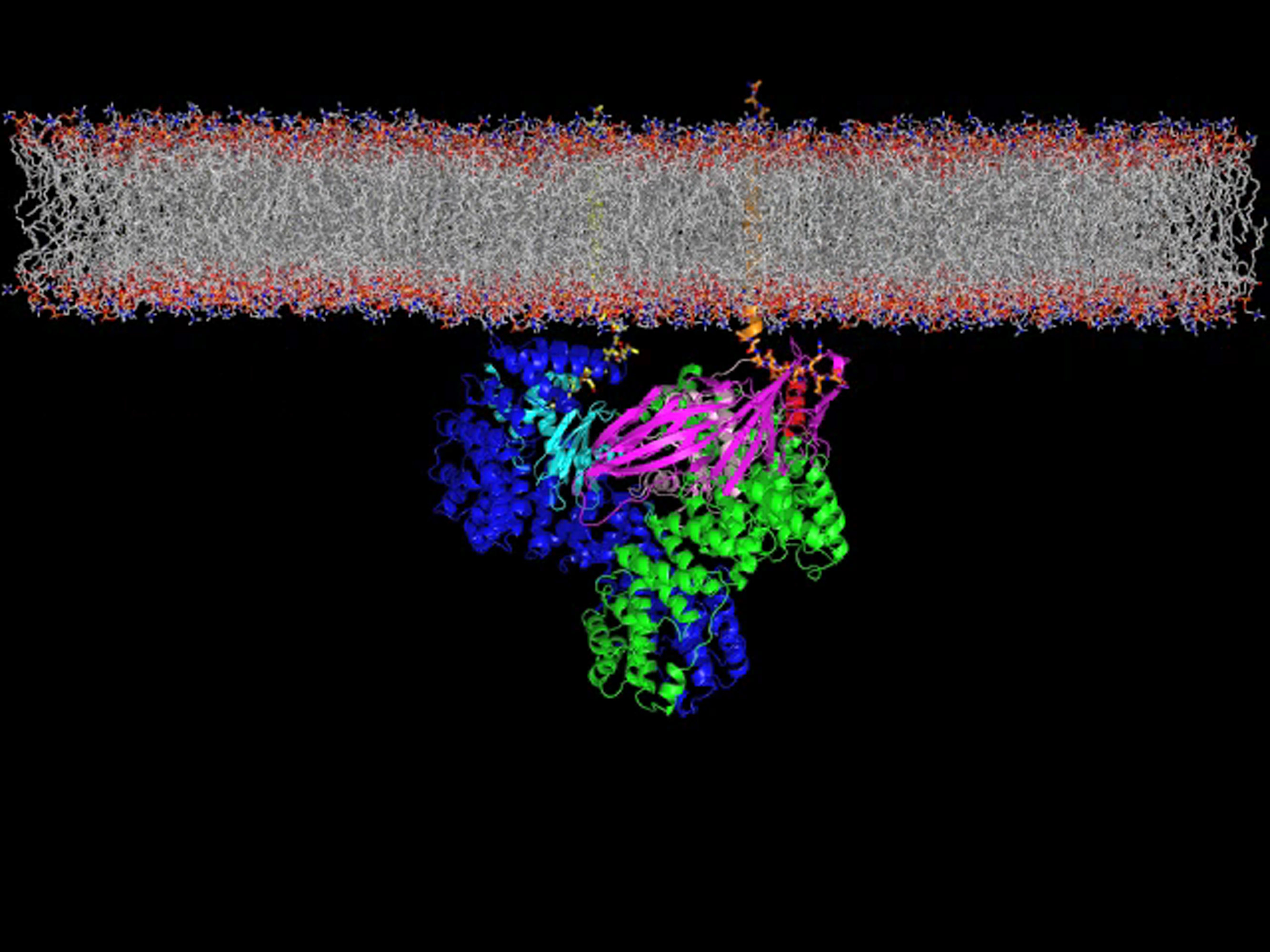

Supplement: Movie S3. Related to Figure 7 — Model of AP2 activation and cargo binding at the plasma membrane. Interpolation between the locked and open conformations was performed using the Rigimol plugin for PyMOL (http://www.pymol.org). The movie was animated using PyMOL. Membrane representation was constructed using a theoretical model (Heller et al., 1993). [file mmc3.jpg]
