# Supplementary material for: A Large-Scale Conformational Change Couples Membrane Recruitment to Cargo Binding in the AP2 Clathrin Adaptor Complex
Source: Cell. 2010 Jun 25;141(7):1220–9. doi: 10.1016/j.cell.2010.05.006 (PMC3655264; doi:10.1016/j.cell.2010.05.006)
Supplement: Document S1. Tables S1 and S2 [file mmc4.pdf]

Table S1. Analysis of open and locked core buried surface areas, related to figure 4

Buried surface areas (to nearest 10 Å ^{2})

Contact		***locked***	***open***
α	σ2	2300	2670
β2	N-μ2	2430	2370
α	β2	1740	1850
α	N-μ2	170	200
σ2	N-μ2	80	80
σ2	β2	630 (430*)	310
C-μ2	N-μ2	380	360
C-μ2	σ2	510	0
C-μ2	α	960	0
C-μ2	β2	840	1560

* The number in brackets is the contribution from the β2 N-terminal residues 4-12 conformation: the figure for the open conformation excludes the pseudo-acidic dileucine sequence. Buried interaction surfaces were calculated and analysed in PISA (Krissinel and Henrick, 2005)

Table S2. Crystallographic data for the open form of AP2, related to Experimental Procedures

**Compound**	**Native**	**TaBr**	**Xe1**	**Xe2**
Resolution range Å (outer bin)	64 – 3.1<br>(3.27 – 3.10)	46 – 5.0<br>(5.27 – 5.0)	75 – 3.5<br>(3.69 – 3.5)	74 – 3.5<br>(3.69 – 3.5)
Beamline	ESRF ID29	ESRF ID29	ESRF ID29	Diamond I02
Number of crystals	1	3	3	1
Wavelength Å	0.873	1.254	1.800	1.771
R_{merge}	0.195 (1.15)	0.223 (0.85)	0.331 (1.8)	0.128 (0.81)
R_{merge} (top intensity bin)	0.075	0.118	0.180	0.046
R_{meas} (within I+/I-)	0.214 (1.26)	0.239 (0.90)	0.351 (1.9)	0.171 (1.08)
R_{pim} (within I+/I-)	0.087 (0.52)	0.084 (0.31)	0.118 (0.64)	0.112 (0.71)
$< <I>)/\sigma(<I>) >$	5.7 (1.3)	9.6 (3.4)	5.9 (1.7)	3.6 (0.9)
Completeness %	96.7 (97.7)	99.8 (100)	100 (100)	93.0 (88.4)
Multiplicity	5.5 (5.5)	16.2 (16.5)	17.5 (17.6)	2.4 (2.3)
Anomalous completeness %	-	99.8 (100)	100 (100)	45.6 (36.7)
Anomalous multiplicity	-	8.0 (8.2)	8.5 (8.4)	1.5 (1.5)
Δ_{anom} correlation (half-datasets)	-	0.484 (0.0)	-0.28 (-0.04)	0.120 (0.08)
Anomalous normal probability slope	-	1.21	0.922	0.916
Anisotropy ΔB Å^2	42	25	23	15
**Phasing (1)**				
Phasing power (Isomorphous/Anomalous)		0.41 / 0.71	0.40 / 0.22	–
Mean Figure of merit (inner resoution bin)	0.10 (0.84)	–	–	–
<FoM> after solvent flattening (68%)	0.82			
**Phasing (2)**				
Phasing power (Isomorphous/Anomalous)		0.38 / 0.67	0.56 / 0.22	0.36 / 0.20
Mean Figure of merit (inner resolution bin)	0.16 (0.83)	–	–	–
<FoM> after solvent flattening (68%)	0.86			
**Refinement**				
Number of reflections (free set)	63145 (3376)			
R / R_{free}	0.197 / 0.264			
Number of atoms	14153			
<B> Å^2	140			
RMS bond length deviation Å (angles °)	0.022 (1.9)			

$R_{merge} = ©_{hl} \mid I_{hl} - <I_h> \mid / ©_{hl} <I_h>$; $R_{meas} = ©_{hl} [n_h/(n_h-1)]^{1/2} I_{hl} - <I_h> \mid / ©_{hl} <I_h>$

$R_{pim} = ©_{hl} [1/(n_h-1)]^{1/2} I_{hl} - <I_h> \mid / ©_{hl} <I_h>$ where n_h is the number of observations of reflection **h**.

Δ_{anom} correlation is the correlation coefficient between anomalous differences calculated for random half-datasets.
