# Supplementary material for: A Large-Scale Conformational Change Couples Membrane Recruitment to Cargo Binding in the AP2 Clathrin Adaptor Complex
Source: Cell. 2010 Jun 25;141(7):1220–9. doi: 10.1016/j.cell.2010.05.006 (PMC3655264; doi:10.1016/j.cell.2010.05.006)
Supplement: Document S2. Article Plus Supplemental Information [file mmc5.pdf]

Cell

# A Large-Scale Conformational Change Couples Membrane Recruitment to Cargo Binding in the AP2 Clathrin Adaptor Complex

Lauren P. Jackson,[1,5] Bernard T. Kelly,[1,5] Airlie J. McCoy,[1] Thomas Gaffry,[2] Leo C. James,[3] Brett M. Collins,[4] Stefan Höning,[2] Philip R. Evans,[3,*] and David J. Owen[1,*]
[1]Cambridge Institute for Medical Research, Department of Clinical Biochemistry, University of Cambridge, Hills Road, Cambridge CB2 0XY, UK
[2]Institute of Biochemistry I and Center for Molecular Medicine Cologne, University of Cologne, Joseph-Stelzmann-Str. 52 50931 Cologne, Germany
[3]Medical Research Council Laboratory of Molecular Biology, Hills Road, Cambridge CB2 0QH, UK
[4]Institute for Molecular Bioscience, The University of Queensland, Brisbane QLD 4072, Australia
[5]These authors contributed equally to this work
*Correspondence: pre@mrc-lmb.cam.ac.uk (P.R.E.), djo30@cam.ac.uk (D.J.O.)
DOI 10.1016/j.cell.2010.05.006

## SUMMARY

The AP2 adaptor complex (α, β2, σ2, and μ2 subunits) crosslinks the endocytic clathrin scaffold to PtdIns4,5$P_2$-containing membranes and transmembrane protein cargo. In the "locked" cytosolic form, AP2's binding sites for the two endocytic motifs, YxxΦ on the C-terminal domain of μ2 (C-μ2) and [ED]xxxL[LI] on σ2, are blocked by parts of β2. Using protein crystallography, we show that AP2 undergoes a large conformational change in which C-μ2 relocates to an orthogonal face of the complex, simultaneously unblocking both cargo-binding sites; the previously unstructured μ2 linker becomes helical and binds back onto the complex. This structural rearrangement results in AP2's four PtdIns4,5$P_2$- and two endocytic motif-binding sites becoming coplanar, facilitating their simultaneous interaction with PtdIns4,5$P_2$/cargo-containing membranes. Using a range of biophysical techniques, we show that the endocytic cargo binding of AP2 is driven by its interaction with PtdIns4,5$P_2$-containing membranes.

## INTRODUCTION

The regulated movement of proteins and lipids between the many cellular membranes in coated vesicular carriers is important for signaling, homeostasis, defining the interactions of cells with their surroundings, and in controlling the glycosylation and proteolytic processing of lumenal and transmembrane proteins. Clathrin-coated vesicles (CCVs) mediate many post-Golgi trafficking routes including internalization from the plasma membrane via clathrin-mediated endocytosis (CME) (Traub, 2009). CCVs have a three-layered structure with an inner membrane layer linked by clathrin adaptors to an outer polymeric clathrin scaffold (Cheng et al., 2007; Fotin et al., 2004). Clathrin adaptors contain a folded membrane-proximal domain, which binds to phosphatidyl inositol polyphosphate (PIP) headgroups and/or Arf GTPases in their membrane-attached, GTP-bound forms, and at least one natively unstructured region, which harbors a clathrin-binding motif (Owen et al., 2004). Transmembrane proteins are generally selected as cargo for incorporation into a CCV through the direct interaction of either widely used, short, linear sequence motifs or covalently attached ubiquitin chains with the membrane-proximal portion of a clathrin adaptor (reviewed in Bonifacino and Traub, 2003; Traub, 2009).

The family of heterotetrameric vesicle coat adaptors comprises APs 1–4 and the β,γ,δ,ζ subcomplex of COPI (Schledzewski et al., 1999). AP2 consists of α, β2, μ2, and σ2 subunits (Figure S1 available online) and is the most abundant endocytic clathrin adaptor (Keen et al., 1981; Pearse and Robinson, 1984). The 70 kDa trunk domains of α and β2, together with the 50 kDa μ2 and the 17 kDa σ2 subunits, form the 200 kDa membrane-proximal core (Collins et al., 2002; Heuser and Keen, 1988). The 30 kDa bilobal C-terminal appendages of α and β2 each possess two sites for binding different motifs found on many other endocytic CCV coat proteins with various accessory/regulatory functions in CCV formation (reviewed in Owen et al., 2004; Schmid and McMahon, 2007). The appendages are connected to the core via long flexible linkers (Zaremba and Keen, 1983) that contain clathrin-binding motifs. The AP2 core is the site of binding to the two widely used endocytic cargo motifs, YxxΦ (where Φ is a hydrophobic residue: I, L, M, F, or V) and [ED]xxxL[LI] or "acidic dileucine" motifs, as well as to the plasma membrane PIP, phosphatidyl inositol-4,5-bisphosphate (PtdIns4,5$P_2$). The AP2 complex therefore acts as a central hub for CCV formation, binding to clathrin, cargo molecules, accessory proteins, and membranes, and these interactions must be under strict spatiotemporal control.

Two structures of the AP2 core have been solved, one with a PIP headgroup analog (inositol hexakisphosphate [$IP_6$]) but

lacking bound YxxΦ or [ED]xxxL[LI] motifs (Collins et al., 2002), and the other in complex with an [ED]xxxL[LI] motif peptide (Kelly et al., 2008). The α and β2 trunk domains are solenoids of stacked α helices, and the N-terminal μ2 domain (N-μ2; residues1–135) and σ2 domain are globular "longin domain" folds. Together, these four domains are arranged into a "bowl," in which the elongated C-terminal μ2 domain (C-μ2) sits (Figure S1). Two positively charged sites that can bind PtdIns4,5$P_2$, one on α and one on μ2, have been identified (Collins et al., 2002; Gaidarov et al., 1996; Rohde et al., 2002) with the basic site on α playing a key role in the initial docking of AP2 onto PtdIns4,5$P_2$ and cargo-containing membranes (Gaidarov et al., 1996; Höning et al., 2005).

The binding site for [ED]xxxL[LI] motifs is located mainly on σ2 (Chaudhuri et al., 2009; Doray et al., 2007; Kelly et al., 2008), whereas YxxΦ motifs bind through a β-augmentation to C-μ2 (Ohno et al., 1995; Owen and Evans, 1998). In the motif-free conformation first shown for AP2 and subsequently for AP1 (Heldwein et al., 2004), both cargo-motif binding sites are blocked by portions of the β2 subunit: β2Tyr6 and β2Phe7 block the [ED]xxxL[LI]-binding site and β2Glu364–β2Val406 (especially β2Val365 and β2Tyr405) block the YxxΦ site (numbering refers to AP2) (Figure S1), and thus we term this the "locked" conformation. In the recent [ED]xxxL[LI] motif-liganded structure, which we term the "unlatched" conformation, the N terminus of β2 is displaced, allowing [ED]xxxL[LI] motif binding to occur. Although in the unlatched form the subunits of the bowl have moved a little relative to each other as compared with the locked form, the YxxΦ motif-binding site remains blocked (Kelly et al., 2008). In both the locked and unlatched structures, the main PtdIns4,5$P_2$-binding site on the α subunit and the [ED]xxxL[LI] motif-binding site are adjacent to each other and located on what was consequently proposed to be part of the AP2 membrane-interacting surface, but the YxxΦ motif-binding site is located on an orthogonal face. It has also been shown that the spacer between the end of a protein's transmembrane helix and a YxxΦ motif need only be seven residues (around 25 Å for a fully extended peptide) (Rohrer et al., 1996). However, in the locked and unlatched structures, the YxxΦ-binding site is 60–70 Å distant from the putative membrane-interacting surface. Taken together, these data suggested that AP2 must undergo a large-scale conformational change in order to allow binding to YxxΦ-containing cargo embedded in a PtdIns4,5$P_2$-containing membrane.

Here we present the structure of a form of AP2 in which both YxxΦ and [ED]xxxL[LI] motif-binding sites are occupied and form a coplanar arrangement with four positively charged patches that are sites for binding the PtdIns4,5$P_2$-rich plasma membrane. Achieving this new conformation and relieving the intramolecular inhibition of the cargo motif-binding sites are facilitated by large changes occurring to the structure of the whole AP2 core.

## RESULTS

In order to compete effectively with the intramolecular blocking of the YxxΦ-binding site and so shift the equilibrium in solution to a YxxΦ-bound conformation, AP2 core was preincubated with a very large (70-fold) molar excess of the TGN38 YxxΦ peptide DYQRLN (35 μM AP2 with 2.5 mM peptide). Crystals of the AP2 complex with bound peptide grew under a number of conditions, but only those grown using mixtures of ammonium sulfate and lithium sulfate showed useful diffraction. These rhombohedral crystals diffracted anisotropically to 3.1 Å resolution in the best direction but little beyond 5 Å in the worst. Experimental phasing was carried out using the $Ta_6Br_{12}^{2+}$ cluster compound and cryo-trapped Xe derivatives, followed by density modification. The bowl of the AP2 core and C-μ2 could be seen in the resulting electron density (Figures 1A and 1B). It was immediately obvious that a large conformational change had occurred, as C-μ2 was no longer located in the center of the bowl but on an orthogonal face (Figures 1E and 1F, compare Figures 1C and 1D). Model building began by placing rigid-body components of the unlatched AP2 structure into the electron density. Electron density was visible for the linker connecting the two domains of μ2, showing that most of it adopted a helical conformation (Figure 2A), and for the YxxΦ peptide (Figure 2B). Surprisingly, electron density was also visible in the [ED]xxxL[LI] motif-binding site on σ2, although no appropriate exogenous ligand had been included in the crystallization experiments. Inspection of the electron density (Figure 2C) suggested that this "phantom" [ED]xxxL[LI] motif was part of the myc-tag sequence (MEQKLI) inserted into a surface loop of μ2 (residues 218–252) at residue 236, given that at low contour levels almost continuous density could be seen extending from the dileucine-binding site to residue 252. This loop was disordered in the locked and unlatched forms. In this "open" form, the myc-tag-containing loop reaches over to a neighboring molecule and forms a vital crystal-packing contact. When the myc-tag was removed from the loop, the resulting protein yielded no diffracting crystals under any crystallization conditions including those used here.

### Conformational Change in the AP2 Bowl

The AP2 bowl can be considered as an extended helical solenoid running continuously from the N terminus of α through the interface between the α and β2 subunits' C termini to a dozen residues short of the N terminus of β2, forming a puckered ring like the seam of a tennis ball. When going from locked to the open form, the AP2 bowl collapses inwards, expelling C-μ2 (Figure 3, Figure S2, and Movie S1 and Movie S2). The collapse brings both lobes of the puckered ring closer together, while the ring dislocates between the α and β2 N termini. The "collapsed" conformation of the bowl is likely to be its lower energy state as a version of the core lacking C-μ2 altogether (μ2-truncated core) also adopts the collapsed conformation (see Extended Experimental Procedures).

The collapse of the AP2 bowl is facilitated by rotations about four "hinge points," two each in α and in β2 (Figure S2). Thus each large subunit can be considered as being composed of essentially three rigid groups. The total buried subunit interfaces for the locked and open forms are 10,040 Å$^2$ and 9700 Å$^2$, respectively. Given that buried surface area is highly correlated with energy and therefore stability (Krissinel and Henrick, 2007), these data suggest that the locked form is more stable and therefore predominates in solution. The major contributions to the buried

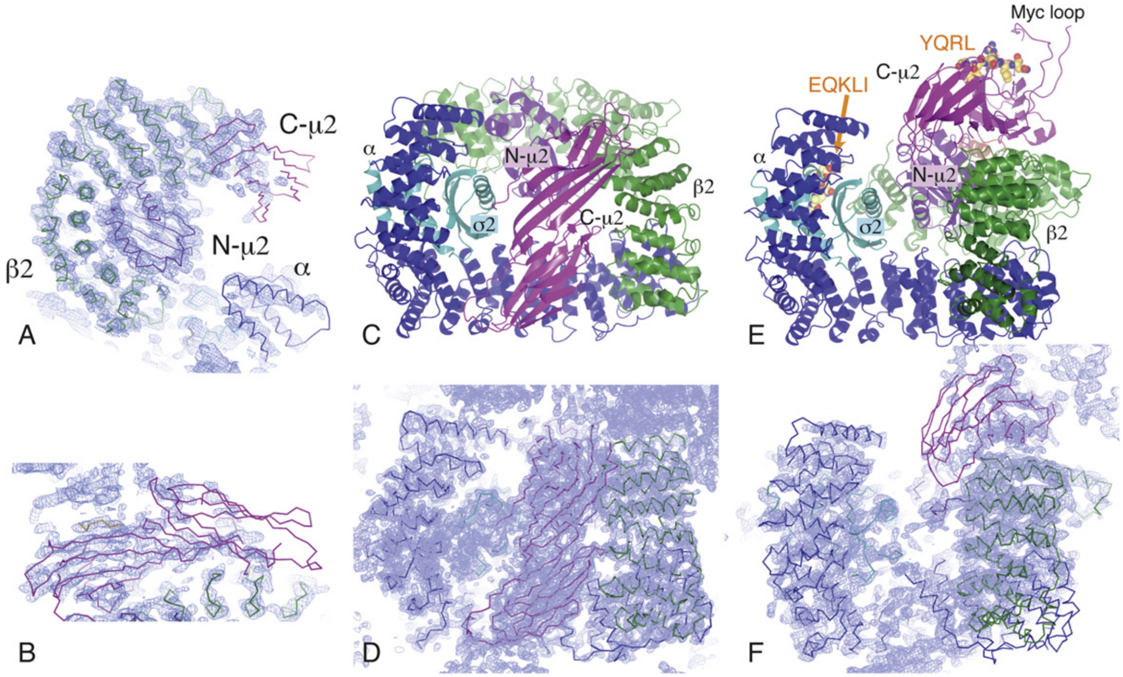


**Figure 1. Structure of the Open Conformation of the AP2 Core**

(A) Part of the experimentally phased, solvent-flattened electron density map with the refined model superimposed.

(B) The experimental electron density map in the region of the C-terminal domain of μ2, showing good density for the first subdomain (left) but very weak density for the poorly ordered second subdomain (right).

(C) An overall view of the whole AP2 core in the locked conformation. The bowl is formed by the helical solenoids of the α (blue) and β2 (green) subunits, together with σ2 (cyan) and the N-terminal domain of μ2 (purple). The C-terminal domain of μ2 (C-μ2) (magenta) is in the center of the bowl. This coloring scheme is used in all subsequent figures.

(D) Experimental electron density for the locked form (Collins et al., 2002), in the same view as (C), showing the C-μ2 in the center of the bowl.

(E) An overall view of the whole AP2 core in the open conformation. C-μ2 is no longer in the bowl, but on the outside of β2, and carries the YxxΦ motif peptide (gold). The myc loop within C-μ2 that reaches into the acidic dileucine motif-binding site on a neighboring molecule in the crystal is labeled, and the site itself is indicated (LI (Myc) peptide). In subsequent pictures this myc loop is omitted for clarity, but the EQKLI sequence is shown in its position on σ2.

(F) Experimental electron density for the new open form, in the same view as (C), showing no density for C-μ2 in the bowl but density in its new position on the outside of the β2 subunit.

subunit interfaces come from the interactions between α and σ2, α and β2, and β2 and N-μ2, which remain largely unaltered during the conformational change (see Table S1 and Figure S3), whereas the major changes in subunit packing are those made by C-μ2 (see below) and the N terminus of β2 being displaced from the [ED]xxxL[LI] motif-binding site on σ2 (Kelly et al., 2008).

## Membrane Binding and the Repositioning of C-μ2

The most spectacular subunit rearrangement, and that of the greatest biological significance, is the movement of C-μ2 relative to the bowl. This can be described geometrically as a screw rotation of C-μ2 by ~129° about its long axis, with a translation of 39 Å (about half its length), relative to N-μ2 (Figure 4A). The trajectory followed by C-μ2 during the actual conformational switching must be greater than this in order to avoid colliding with β2 (see Movie S3). The subunit contacts made by C-μ2 change completely between the locked and open conformers (Figure 4B). All contacts between C-μ2 and α and between C-μ2 and σ2 are lost, and although the surface area buried between C-μ2 and β2 doubles (see below and Figure S3 and Table S1), the total subunit interface area buried by C-μ2 in the open form drops by around 1200 $Å^2$ as compared with the locked form. However, this loss of buried surface area is partly compensated for by the 800 $Å^2$ surface area buried by the μ2 linker binding back onto the rest of the core (see below, Figure 4 and Table S1).

In the locked and unlatched forms, the μ2 linker (residues 130–158), which contains the AAK1 (α-appendage binding kinase) catalyzed phosphorylation site at μ2Thr156 (Conner and Schmid, 2002), is disordered, thus making it ideally suited to recognition by the protein kinase. This phosphorylation is important for AP2 function in vivo because mutating the phosphorylation site inhibits transferrin uptake (Motley et al., 2006; Semerdjieva et al., 2008). In vitro, μ2Thr156 phosphorylation enhances the binding of AP2 to endocytic motifs (Höning et al., 2005; Olusanya et al., 2001; Ricotta et al., 2002) presumably by driving the equilibrium toward the cargo-binding-competent open form. In the open form the linker forms a four-turn helix, which packs in a trough lined by residues from β2, N-μ2, and C-μ2 (Figure 4 and Figure S3). In the locked form this trough was present but unoccupied. It is possible that the μ2Thr156 phosphorylation event further stabilizes this helical conformation

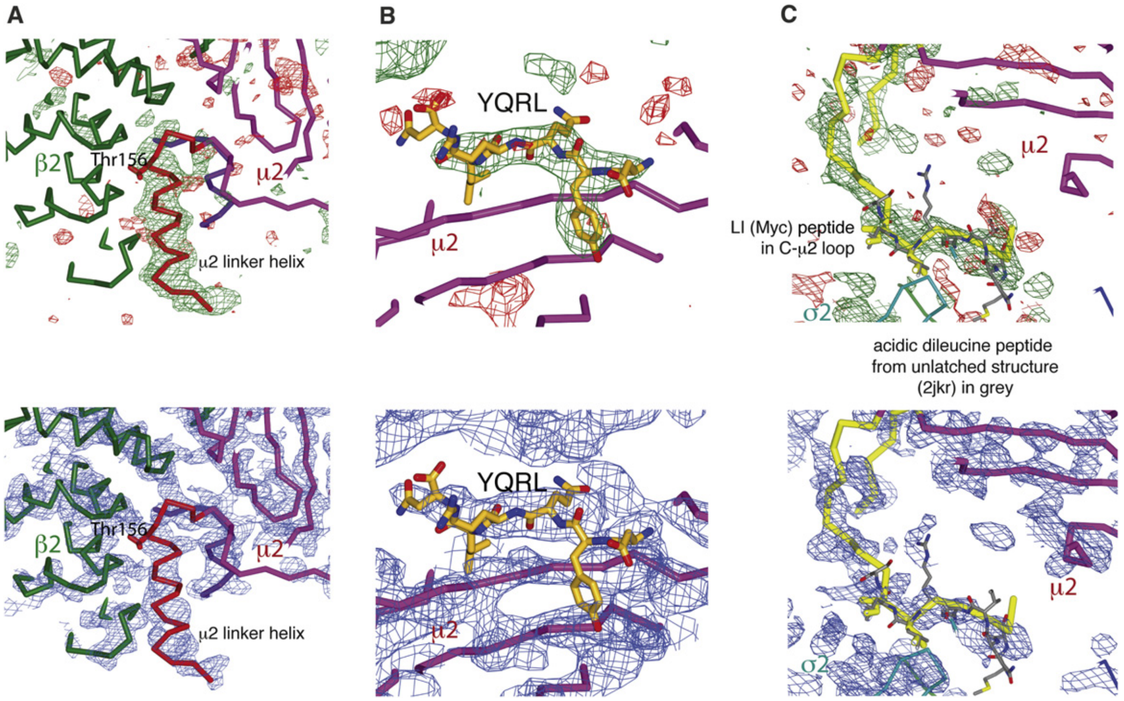


**Figure 2. New Structural Features Not Found in the "Locked" Structure**

The top panels are "omit" maps, mFo-DFc difference maps calculated by omitting part of the structure, randomly displacing all the atoms a little and then refining, using the experimental phases as restraints. Omitted residues are colored red (linker) or yellow (YxxΦ peptide and dileucine peptide mimic). The lower panels show the solvent-flattened experimental map.

(A) The μ2 linker folds into a helix lying in a groove between N-μ2 and β2: the side chain of Thr156, which can be phosphorylated, is shown.

(B) The YxxΦ motif peptide is bound to the C-μ2 domain, in the equivalent position to that found on the isolated C-μ2 domain (Owen and Evans, 1998).

(C) Electron density in the acidic dileucine peptide-binding site on σ2 is linked to C-μ2 across a crystal contact and has been interpreted as part of the myc-tag EQKLI. The peptide QIKRLL from the unlatched structure (gray) is shown in its position relative to σ2.

of the linker by interacting with positively charged residues in the vicinity of the trough, especially β2Arg138 and β2Lys139 (Figure S3). Unfortunately all attempts to crystallize the phosphorylated form of AP2 have so far failed, and attempts to mutate candidate residues resulted in poorly expressed, aggregation-prone complexes. Further, an attempt to mimic the phosphorylation event by mutating μ2Thr156 to glutamate was also unsuccessful: μ2Thr156Glu core shows 4-fold weaker binding to YxxΦ/PtdIns4,5$P_2$-containing liposomes than wild-type core and 20-fold less binding than phosphorylated AP2 core (S.H., unpublished data).

The result of the relocation of C-μ2 is that all three previously biochemically confirmed ligand-binding sites (those for PtdIns4,5$P_2$, YxxΦ motifs, and [ED]xxxL[LI] motifs) become coplanar on a surface of AP2 and are thus suitably arranged for contacting the various signals in the context of the plasma membrane. There are three other regions of highly positive electrostatic potential on this surface in addition to the α subunit PtdIns4,5$P_2$-binding site (Figure 5). The first is formed by basic residues from the N terminus of β2 (Lys5, Lys12, Lys26, Lys27, Lys29, Lys36). A version of the AP2 core in which these residues are mutated to glutamate (β2$^{PIP-}$Core) cannot be recruited to PtdIns4,5$P_2$-containing membranes, similar to the version of the AP2 core harboring mutations in the PtdIns4,5$P_2$-binding site on α (α$^{PIP-}$Core) (Figure 5 and Höning et al., 2005). The second and third basic regions are on the surface of C-μ2. The second basic region (Lys330, Lys334, Lys350, Lys352, Lys354, Lys356, Lys365, Lys367, Lys368, Lys373) was identified as a putative PtdIns4,5$P_2$-binding site (Collins et al., 2002; Rohde et al., 2002). Mutation of five lysine residues to glutamate in this region (5K>E) slightly weakens the binding of AP2 cores to PtdIns4,5$P_2$-containing membranes (dissociation constant [$K_D$] 11 μM instead of 7 μM). In the third region (Lys167, Arg169, Arg170, Lys421), the substitution of three basic residues (KRR>E) has little effect on AP2 binding to PtdIns4,5$P_2$-containing membranes. However, combining the mutations (μ2$^{PIP-}$Core) results in an AP2 core with a 4-fold reduction in binding to PtdIns4,5$P_2$-containing liposomes (Figure 5). All mutant forms of the AP2 core were correctly folded as judged by identical levels of expression, incorporation into complexes, and CD spectra (data not shown). These data suggest that initial membrane recruitment of AP2 occurs mainly through the α and β2 PtdIns4,5$P_2$-binding sites with the basic surface on C-μ2 playing an auxiliary role. However, we propose that the C-μ2 basic regions are key to driving the opening of AP2 by the electrostatic attraction of C-μ2 to the PtdIns4,5$P_2$ membrane, as μ2$^{PIP-}$Core mutant shows strongly inhibited (20-fold) binding to PtdIns4,5$P_2$/YxxΦ liposomes. The binding of the μ2$^{PIP-}$Core mutant to PtdIns4,5$P_2$/[ED]xxxL[LI] liposomes is only reduced by 3-fold, suggesting that acidic dileucine motif binding does

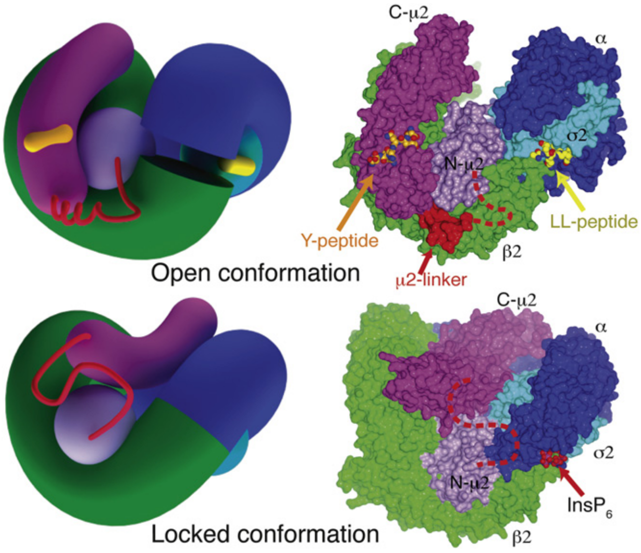


**Figure 3. The Conformational Change in AP2 on Cargo Binding**
Schematic (left) and surface (right) representations of the open (upper) and locked (lower) conformations. In the transition from the locked to the open structure, the puckered ring formed from α and β2 narrows and splits between the N termini, and C-μ2 emerges from its bowl and rotates roughly about its long axis. σ2 and N-μ2 remain fixed to α and β2, respectively.

not strictly need C-μ2 to bind to the membrane, only its displacement from the bowl.

## Endocytic Motif Binding

Once attached to and stabilized on the membrane through binding multiple PtdIns4,5$P_2$ molecules, the open form of the complex can be further stabilized by the binding of membrane-embedded YxxΦ and [ED]xxxL[LI] cargo motifs in their respective sites, to which there is now unrestricted access for cargoes embedded in the membrane (Figure 6). Our motif-liganded structure suggests that both motif-binding sites could be occupied simultaneously. We would therefore predict that AP2 would exhibit tighter binding to a membrane harboring *both* endocytic motifs than to one with either motif alone, due to the avidity effect of simultaneous binding. This is indeed the case (Figure 5 and Figure S4). However, whether two different cargoes can bind simultaneously to a single AP2 in vivo will depend on steric clashes between their extracellular domains, the flexibility of their juxtamembrane regions, and the ratios of clathrin:AP2: cargo in a given CCV.

The open form structure suggests that a single cargo protein containing *both* motifs could, in principle, bind with both motifs engaged by the same AP2 complex, but only if the motifs were separated by at least 65–70 Å (corresponding to a spacing of around 25 residues), but this cannot formally be demonstrated in our assay system. Inspection of a database of type I and II membrane proteins (M. Robinson, personal communication) showed that in only a very few cases do both motifs occur separated by at least 20 residues within their unstructured regions, and even in these cases (CIMPR [Chen et al., 1993], furin [Voorhees et al., 1995], vGlut-1 [Kim and Ryan, 2009], and LRP1[Li et al., 2001], which have complex trafficking itineraries) it appears that one motif is used mainly for internalization and the other for intracellular trafficking.

A question that naturally arises is, if this open structure is energetically stable, is the locked, non-cargo-binding form biologically relevant, i.e., is it the conformation of AP2 in solution? This seems likely, as in the absence of ligands, the locked conformation buries a larger total surface area and is therefore more stable than the open form; furthermore, the locked conformation has also been seen independently in AP1 (Heldwein et al., 2004). Two further lines of evidence support the hypothesis that AP2 adopts the locked conformation in solution. First, in a surface plasmon resonance (SPR) assay, preincubation of AP2 cores with an excess of YxxΦ motif-containing peptide has no effect on the association of AP2 with PtdIns4,5$P_2$ and YxxΦ motif-containing liposomes, whereas preincubation of isolated C-μ2 with an excess of the same peptide is sufficient to abolish binding to the same liposomes (Figure S5). Second, equilibrium fluorescence anisotropy experiments in which a fluorescein-labeled YxxΦ motif-containing peptide was titrated with C-μ2 or with AP2 core reveal that isolated C-μ2 binds the peptide with a $K_D$ of ~1.9 μM, whereas the AP2 core exhibits no detectable binding (Figure S5).

The most likely candidate for driving and subsequently stabilizing the conformational change to a cargo-binding-competent open form is interaction with multiple PtdIns4,5$P_2$ molecules arranged in a roughly planar fashion in the membrane. This is supported by three lines of evidence. First, if no PtdIns4,5$P_2$ is included in liposomes that contain both YxxΦ and [ED]xxxL[LI] motifs, there is no detectable binding of AP2 (Figure 6). Second, mutation of any one of the basic PtdIns4,5$P_2$-binding patches on α or β2, or the combined pair on μ2, strongly inhibits binding to membranes containing PtdIns4,5$P_2$ and YxxΦ sorting motifs (Figure 5 and Figure S4), i.e., high-affinity binding to YxxΦ-containing membranes requires multiple simultaneous PtdIns4,5$P_2$-binding events on α, β2, and μ2 that can only occur when AP2 is in its open conformation. Finally, in the fluorescence anisotropy assay measuring the equilibrium association of AP2 with fluorescein-labeled YxxΦ peptide, the binding of AP2 was strongly promoted by the presence of the polyanionic heparin (~50-mer), which, with multiple negative charges disposed along a single large molecule, can mimic the arrangement of charges in a membrane. The binding for AP2 to free YxxΦ peptide increases from undetectable levels to a $K_D$ of ~3.4 μM in the presence of heparin, which is a similar strength of binding to that of isolated C-μ2 for the same peptide, $K_D$ ~1.9 μM (Figure S5).

To explore the nature of AP2 activation further we used polarized fluorescence stopped-flow spectrophotometry to follow the pre-steady-state kinetics. Isolated C-μ2 and AP2 preincubated with heparin both display rapid binding to YxxΦ peptide (Figure S5), with fast on-rates (~$10^6$ $M^{-1}$ $s^{-1}$) and off-rates of ~2 $s^{-1}$. The kinetically derived dissociation constants of C-μ2 and heparin-incubated AP2 (0.5 μM and 3.5 μM, respectively) closely match equilibrium measurements, confirming that interaction occurs in a single step. In contrast, YxxΦ binding by AP2 in the absence of heparin occurs extremely slowly (Figure S5). Heparin-activated AP2 binds with a relaxation time

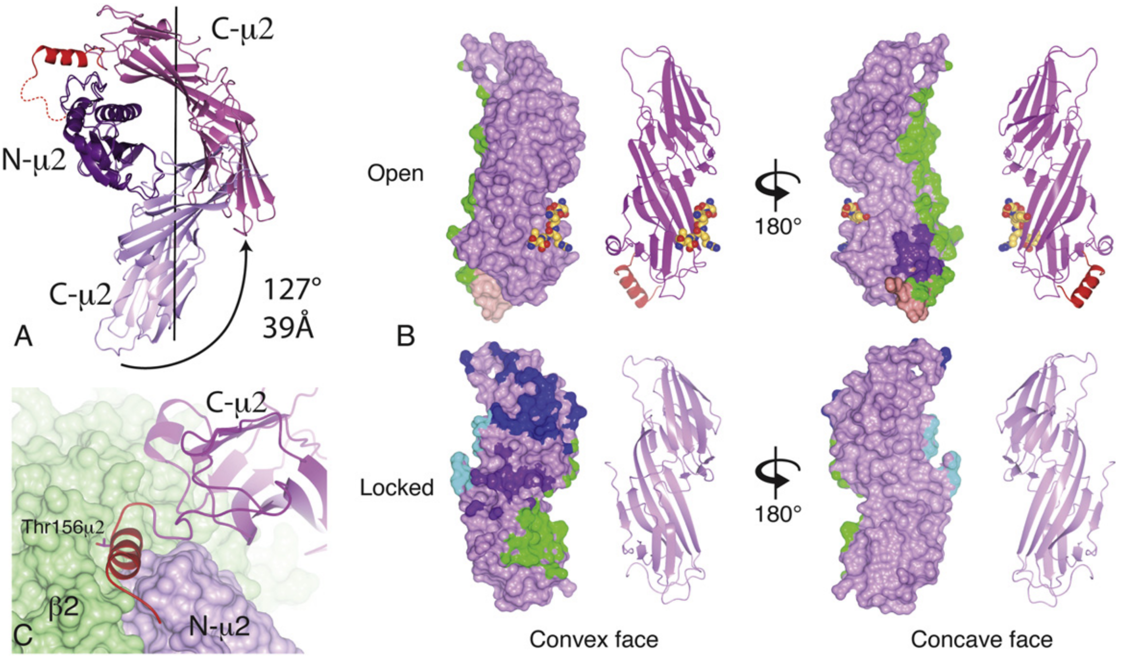


**Figure 4. Changes in the Positioning and Interactions of C-μ2**

(A) In the transition from the locked (pale mauve ribbons) to the open structure (dark mauve), C-μ2 rotates relative to N-μ2 by 127° around an axis (black line) roughly along its long axis with a translation of 39 Å along the axis. The linker helix is shown red.

(B) C-μ2 makes completely different contacts with other subunits in the two structures. Two reversed views of the C-μ2 surfaces are shown, colored mauve (with the linker helix pink), and by their contacts with the other subunits: α blue, β2 green, σ2 cyan, and N-μ2 purple. The YxxΦ motif peptide is shown on the open structure.

(C) The μ2 linker helix lies in a shallow groove between β2 and N-μ2, with the μ2Thr156 residue that can be phosphorylated partly buried.

(a measure of the time in which binding occurs) of 0.056 s, whereas in the absence of heparin this reaction slows ~1000-fold to >60 s (Figure 6). The dramatic difference in kinetics can only be explained by a slow rate-determining reaction that precedes the bimolecular binding step. As the stoichiometry of the AP2:YxxΦ interaction is known to be 1:1, the preceding slow step must represent a pre-equilibrium isomerization to a binding-competent isomer. Calculation of kinetic rate constants (data not shown) gives a derived $K_D$ for AP2:YxxΦ of ~4 mM. The difference between the affinities of AP2 in the presence or absence of heparin suggests that, at equilibrium, >99.9% of non-membrane-bound AP2 is in a locked or inactive conformation. These data show that AP2 is thus unable to associate promiscuously in the cytoplasm with proteins containing trafficking motifs.

## DISCUSSION

The striking conformational change between the locked and the open, ligand-bound active forms of AP2 described here completely remodels the domain arrangement of the heterotetramer. Based on the known AP2 core structures, we propose the following scenario for the activation of AP2 for cargo binding on the plasma membrane. The model assumes that there are two main conformers of AP2, one locked and one open, that are in equilibrium with each other in solution, but that the equilibrium lies heavily in favor of the closed form in the absence of membrane interaction. By characterizing the pre-steady-state interaction of AP2 with a YxxΦ peptide, we have provided direct evidence that AP2 isomerizes between inactive ("locked") and active ("open") isomer forms and shown that >99.9% of non-membrane-bound AP2 in the cell will be in the "locked" conformation. The "activated" form of AP2 binds YxxΦ peptide with a rapid association rate similar to that of isolated C-μ2. This is what we would expect from our structure because the YxxΦ-binding site on C-μ2 is now unobstructed by any part of the complex. Taken together with the coplanar arrangement of all the ligand-binding sites, from which we would predict effects subsequently confirmed by our mutagenesis data (Figure 5), it seems reasonable to equate our open structure with the "activated" form of AP2 on the plasma membrane.

The first step in AP2 activation is its recruitment onto the plasma membrane, primarily by binding through the basic patches on α or β2 to PtdIns4,5$P_2$, which in vitro has an apparent $K_D$ of 7–8 μM (Höning et al., 2005; Figure 5). Once attached to the membrane via α and β2, the electrostatic attraction and subsequent binding of C-μ2 to the high local concentration of PtdIns4,5$P_2$ causes AP2 to adopt the open conformation. The μ2 linker can now be buried in a helical conformation in the slot formed between N-μ2 and β2, again stabilizing the open form. Further stabilization may be achieved through phosphorylation of μ2Thr156 by AAK1, resulting in the apparently tighter binding of μ2Thr156-phosphorylated AP2 to cargo-containing membranes (Höning et al., 2005; Ricotta et al., 2002).

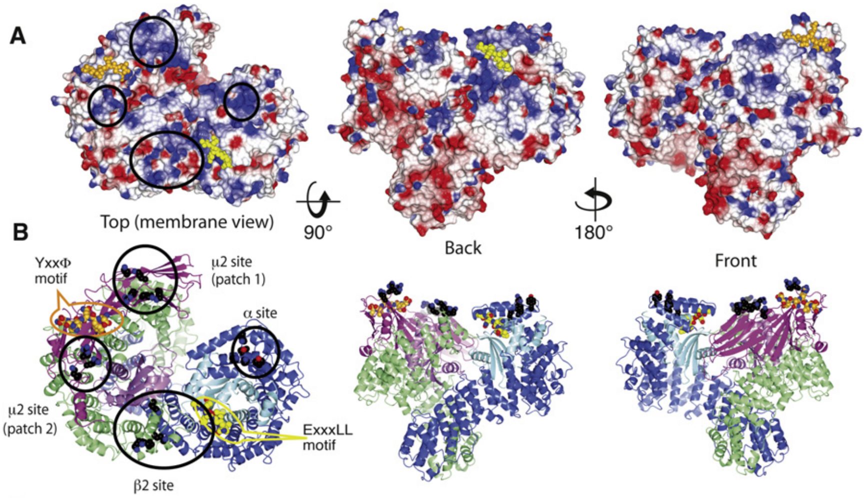


C

| $10\%PtdIns4,5P_2$ + | $K_D(\mu M)$ | 1%TGN38 $K_D(\mu M)$ | 1%CD4P $K_D(\mu M)$ | 0.5%TGN38 + 0.5%CD4 $K_D(\mu M)$ |
|---|---|---|---|---|
| Wild type Core | 7.0 | 0.32 | 0.82 | 0.09 |
| $\alpha$ $^{PIP-}$ Core | -/- | >30 | -/- | >30 |
| $\beta2$ $^{PIP-}$ Core | -/- | 20 | -/- | >30 |
| $\mu2$ $^{PIP-}$ Core | 30 | 6.2 | 2.5 | 0.28 |
| $\mu2$ $^{5K>E}$ Core | 11 | 0.52 | 0.95 | ND |
| $\mu2$ $^{KRR>E}$ Core | 7.5 | 0.33 | 0.85 | ND |

**Figure 5. The Membrane Interaction Surface of AP2**

(A) Three views of the surface colored by electrostatic potential (contoured from −0.5 V [red] to +0.5 V [blue]). Left: top view of positively charged membrane-binding face. Center and right: back and front side views. The two peptides are shown in gold (Y-peptide) and yellow (LL-peptide) and the mutated basic patches are indicated by black circles.

(B) Equivalent ribbon representations, with mutated patches of Lys and Arg residues on α, β2, and C-μ2 that affect membrane binding shown in black.

(C) Equilibrium binding constants for the binding of wild-type and $PtdIns4,5P_2$-binding site mutants of AP2 cores to liposomes displaying the YxxΦ motif of TGN38 or the dileucine sorting signal of CD4, or both signals, displayed in $PtdIns4,5P_2$-containing liposomes as determined by SPR. Values were calculated from rate constants obtained using five different concentrations of AP2 ranging from 50 nM to 1 μM. Sample sensorgrams, corrected for nonspecific binding to PC/PE liposomes and obtained using concentrations of AP2 ranging from 100 nM to 20 μM are shown in Figure S4.

The initial dislocation of C-μ2 from its site on the bowl causes the bowl to relax toward the lower-energy conformation present in the open and μ2-truncated forms of the AP2 core (Extended Experimental Procedures). The most important functional effect of this change in the conformation of the bowl is to cause the β2 subunit to move with respect to the two peptide ligand-binding sites such that it is no longer able to block either. The β2 subunit can therefore be considered as the latch that in solution blocks both peptide-binding sites thus rendering them unusable. Once the motif-binding sites are unblocked, they can then bind to any YxxΦ or [ED]xxxL[LI] motifs in their vicinity, which, because AP2 is held against the membrane, will be those on transmembrane protein cargo. The energy liberated on cargo binding results in the further stabilization of AP2. The AP2 complex is now tightly attached to the membrane via multiple cargo and phospholipid headgroup interactions (apparent $K_D$ around 90 nM; Figure 7; Movie S3).

This model is in agreement with the stabilities of the various AP2 structures as inferred from their buried subunit interfaces (Krissinel and Henrick, 2007). In solution the locked form is more stable (10040 Å$^2$ of buried subunit interface) than the open form (9700 Å$^2$). However, when cargo binding is taken into account, the buried interface area of the open form rises to 10590 Å$^2$ (not including bound lipids), and so the open form becomes the more stable in the presence of a $PtdIns4,5P_2$- and cargo-containing membrane. This two-state model does not exclude the existence of intermediates, such as the [ED]xxxL[LI]-liganded "unlatched" structure (Kelly et al., 2008). However, such a conformer is likely to be short-lived, as C-μ2 will be strongly attracted to the $PtdIns4,5P_2$ of the membrane and should rapidly complete the final stages of the full conformational change to the open form, which can then bind to any available YxxΦ-containing cargo.

Recent live-cell imaging studies (Loerke et al., 2009; Saffarian et al., 2009) have revealed the presence of three types of AP2/clathrin-positive structures at the cell's limiting membrane: those that abort in around 5 s (early abortive), those that abort within 15 s (late abortive), and those that are endocytosed at around 100 s. The model presented here is in line with these findings. The early abortive structures correspond to the situation where AP2 transiently docks to the plasma membrane but fails to undergo the activating conformational change, perhaps because the local concentration of $PtdIns4,5P_2$ is insufficient (Figure 7, lefthand image). The endocytically productive class of structure is that in which AP2 opens and binds to cargo (Figure 7, righthand image). The late-aborted structures therefore correspond to the situation where sufficient $PtdIns4,5P_2$ is present to drive the conformational change (Figure 7, center image) but insufficient cargo is available to further stabilize the binding of AP2 to the membrane. As we would predict from this assignment of states to conformations of our structural model, Schmid and colleagues have shown that overexpressing YxxΦ-containing cargo converts the late-abortive class of clathrin-coated structures to endocytically productive ones. This would be caused by the high concentration of YxxΦ motifs "shifting" the equilibrium between open and closed forms even further toward the open form.

Recent work has suggested that Arf6 plays a role in recruiting AP2 to the plasma membrane (Paleotti et al., 2005), despite the observation that $PtdIns4,5P_2$ is necessary and sufficient to

efficiently recruit AP2 to the membrane. There is, however, little doubt that AP1, AP3, AP4, and COPI are all recruited to their respective membranes primarily through interactions between their large subunits and GTP-bound, membrane-associated Arf1 (Austin et al., 2000; Boehm et al., 2001; Spang et al., 1998). The high degree of structural homology between the four APs and the β,γ,δ,ζ subcomplex of COPI (Schledzewski et al., 1999) suggests that the same gross conformational change that facilitates strong membrane attachment and cargo binding in AP2 will occur in all family members. In the case of AP2 the conformational change is driven by PtdIns4,5P$_2$ (although Arf6 may play a role), but in the case of the other AP family members the change must be driven by Arf1GTP. The most obvious way in which Arf1GTP could shift the equilibrium from the closed to the open state is by binding to and thus stabilizing only an open, cargo-binding-competent conformation very similar to that presented here for AP2. This model predicts that Arf1GTP and cargo binding would be synergistic, and this has indeed been shown to be the case for Arf1 (Baust et al., 2006; Lee et al., 2008).

In summary, we have determined a fully ligand-bound form of AP2, which by comparison with our previous structures shows that the complex has undergone a complicated series of large-scale subunit-repositioning events. The functional need for this massive subunit rearrangement is to allow cargo binding to be coupled to PtdIns4,5P$_2$-containing membrane attachment so as to prevent inappropriate recognition of YxxΦ and [ED]xxxL[LI] sequences on cytoplasmic proteins by AP2 when it is free in the cytosol. The unblocking of both motif-binding sites is elegantly coordinated through the use of different parts of the same β2 subunit to block simultaneously the two separate endocytic motif-binding sites.

## EXPERIMENTAL PROCEDURES

### Structure Determination

Recombinant AP2 cores were made as in Collins et al. (2002). Crystals of the open form were grown from a mixture of AP2 cores (7 mg/ml) with the DYQRLN peptide derived from TGN38 (2 mg/ml) by hanging drop vapor diffusion against a reservoir containing 0.7 M Lithium sulfate, 0.7 M ammonium sulfate, and 200 mM sodium citrate (pH 7.4), 5 mM DTT. Crystals were cryoprotected in mother liquor augmented with 20% glycerol and 3 mg/ml TGN38 peptide, and all data were collected on at 100K at beamline ID29 at ESRF. Crystals were of space group R3 with unit cell dimensions a = 255 Å, c = 157 Å. Diffraction from all crystals was severely anisotropic, extending at best to around 3.1 Å resolution in the a-b plane, but not much beyond 5 Å resolution along c. The structure was solved by multiple isomorphous replacement with anomalous scattering experimental phasing using cryo-trapped Xe (10 atmospheres of pressure for 1 min) and crystals soaked in the $Ta_6Br_{12}^{2+}$ cluster compound (Table S2). The structure was built using a combination of real-space molecular placement and reciprocal space molecular replacement guided by the experimental electron density. The structure was refined using experimental phase restraints. For a full description of the crystallographic methods and structure validation, see the Extended Experimental Procedures.

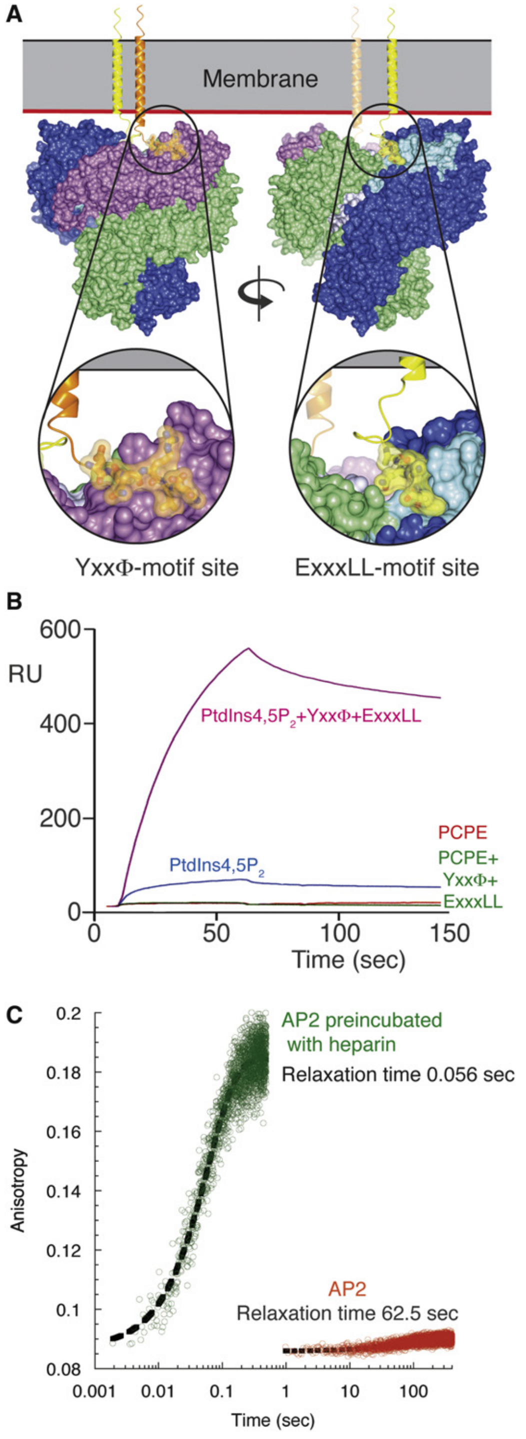


**Figure 6. Interaction of AP2 Cores with YxxΦ and Acidic Dileucine Motifs**

(A) The two motif-binding sites for YxxΦ (gold) and acidic dileucine (yellow) are both freely accessible when AP2 is in the open conformation on the membrane. The peptides are shown attached to a modeled transmembrane helix.

(B) SPR sensorgrams of binding of AP2 core to liposomes with various compositions as indicated showing that there is no detectable binding to liposomes containing both YxxΦ and [ED]xxxL[LI] motif peptides if PtdIns4,5P$_2$ is not present.

(C) AP2 (30 μM orange) or AP2 preincubated with heparin (15 μM green) were rapidly mixed with fluorescein-labeled YxxΦ peptide in a stopped-flow spectrometer and the change in anisotropy upon binding was measured. The anisotropy change was fitted to a single exponential function to obtain relaxation times.

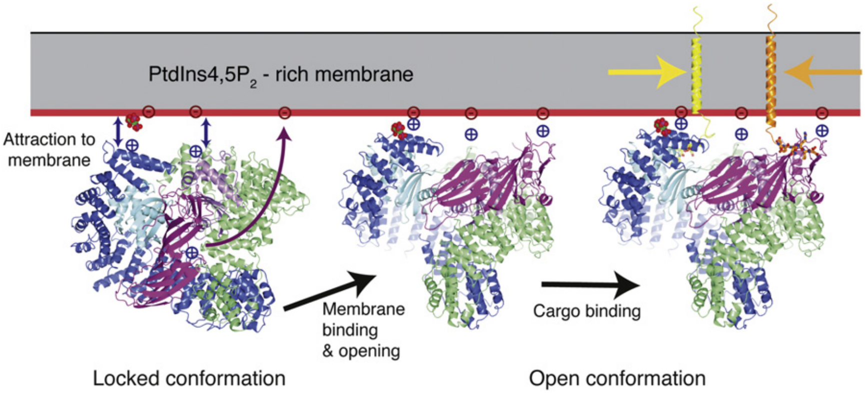


**Figure 7. Membrane-Induced Conformational Switching of AP2 for Cargo Binding**
AP2 in the locked conformation (left) is attracted to the PtdIns4,5$P_2$ headgroups in the membrane through the $\alpha$ and $\beta$2 subunits. The conformational change to the open form (center) is triggered by the electrostatic attraction of C-$\mu$2 to the membrane, which can then bind membrane-embedded cargo motifs (right). An animation is presented in Movie S3.

### Surface Plasmon Resonance Biosensor Experiments

Recombinant AP2 cores, mutants thereof, and C-$\mu$2 were probed for binding to liposomes captured on the L1 surface of a SPR biosensor (BIAcore 3000 and T100). Recording of the interaction and data evaluation were done as described previously (Höning et al., 2005; Kelly et al., 2008). In the competition experiments, AP2 cores and C-$\mu$2 were incubated with the indicated concentrations of TGN38 or CD4 sorting signal peptides for 15 min at room temperature prior to the biosensor experiment, during which binding to PtdIns4,5$P_2$-Yxx$\Phi$-containing liposomes was recorded.

### Equilibrium Fluorescence Anisotropy Measurements

The increase in fluorescence polarization anisotropy upon binding of a large molecule such as AP2 or C-$\mu$2 to a fluorescent peptide was employed as a measure of binding. Further details are described in the Extended Experimental Procedures. Briefly, a peptide encoding the TGN38 Yxx$\Phi$ motif (sequence ASDYQRL) and modified at its N terminus with fluorescein (Sigma-Genosys) was used in all equilibrium binding titration experiments at a concentration of 20 nM. Fluorescence anisotropy was measured using a PheraStar Plus plate reader (BMG Labtech) with increasing pseudo-first order concentrations of AP2, C-$\mu$2, or $\mu$2-truncated core. Where used, polymeric heparin ($\sim$50 subunits) (Rovi Laboratories) was added to 500 $\mu$M concentration. Binding curves (Figure S7) were fitted to a single-site binding model to estimate $K_D$. The $\mu$2-truncated core, lacking the C-$\mu$2 subdomain and therefore unable to bind the Yxx$\Phi$ motif, was used to determine the level of nonspecific background binding (manifested as a linear increase in anisotropy). Without the addition of polymeric heparin, AP2 showed no specific binding compared to $\mu$2-truncated core (Figure S7B).

### Stopped-Flow Polarized Fluorescence Spectrophotometry

Pre-steady-state interaction of a fluorescein-conjugated Yxx$\Phi$-containing peptide with C-$\mu$2 and AP2 was performed using a dual-channel fluorescence TgK single-mix SF-61SX2 stopped-flow spectrometer. A collimated excitation beam at 494 nm passed through a calcite prism polarizer was used to excite 1 $\mu$M Yxx$\Phi$-containing peptide. Parallel and perpendicular polarized fluorescence was measured on independent photomultipliers fitted with 515 nM glass filters from which the fluorescence anisotropy was calculated. Relaxation times were determined for a range of $\mu$M C-$\mu$2 and AP2 concentrations at pseudo-first order excess and used to determine kinetic rate constants (further details are given in the Extended Experimental Procedures). For heparin experiments, AP2 was preincubated for 30 min with 200 $\mu$M heparin.

## ACCESSION NUMBERS

Coordinates have been deposited in the Protein Data Bank with PDB ID 2xa7.

## SUPPLEMENTAL INFORMATION

Supplemental Information includes Extended Experimental Procedures, five figures, two tables, and three movies and can be found with this article online at doi:10.1016/j.cell.2010.05.006.

## ACKNOWLEDGMENTS

We would like to thank the staff of ID29 at the ESRF, especially Elspeth Gordon, and beamline staff at Diamond Lightsource, especially Katherine McAuley and Dave Hall. We also thank Maria José Sánchez Barrena for assistance in data collection, Meredith Roberts-Thompson for assistance with fluorescence anisotropy measurements, and Margaret Robinson for access to the transmembrane protein database. We thank Lesley McKeane for creating part of Figure 3. D.J.O., L.P.J., and B.T.K. are supported by a Wellcome Trust Senior Fellowship to D.J.O.; T.G. and S.H. are supported by the German Science Foundation (SFB 635, TP3, SFB 670 TP7) and the Center for Molecular Medicine Cologne (TP C3); and B.M.C. is supported by a Career Development Award from the National Health and Medical Research Council (NHMRC), Australia.

Received: September 28, 2009
Revised: March 8, 2010
Accepted: April 27, 2010
Published: June 24, 2010

## REFERENCES

Austin, C., Hinners, I., and Tooze, S.A. (2000). Direct and GTP-dependent interaction of ADP-ribosylation factor 1 with clathrin adaptor protein AP-1 on immature secretory granules. J. Biol. Chem. *275*, 21862–21869.

Baust, T., Czupalla, C., Krause, E., Bourel-Bonnet, L., and Hoflack, B. (2006). Proteomic analysis of adaptor protein 1A coats selectively assembled on liposomes. Proc. Natl. Acad. Sci. USA *103*, 3159–3164.

Boehm, M., Aguilar, R.C., and Bonifacino, J.S. (2001). Functional and physical interactions of the adaptor protein complex AP-4 with ADP-ribosylation factors (ARFs). EMBO J. *20*, 6265–6276.

Bonifacino, J.S., and Traub, L.M. (2003). Signals for sorting of transmembrane proteins to endosomes and lysosomes. Annu. Rev. Biochem. *72*, 395–447.

Chaudhuri, R., Mattera, R., Lindwasser, O.W., Robinson, M.S., and Bonifacino, J.S. (2009). A basic patch on alpha-adaptin is required for binding of human immunodeficiency virus type 1 Nef and cooperative assembly of a CD4-Nef-AP-2 complex. J. Virol. *83*, 2518–2530.

Chen, H.J., Remmler, J., Delaney, J.C., Messner, D.J., and Lobel, P. (1993). Mutational analysis of the cation-independent mannose 6-phosphate/insulin-like growth factor II receptor. A consensus casein kinase II site followed by 2 leucines near the carboxyl terminus is important for intracellular targeting of lysosomal enzymes. J. Biol. Chem. *268*, 22338–22346.

Cheng, Y., Boll, W., Kirchhausen, T., Harrison, S.C., and Walz, T. (2007). Cryo-electron tomography of clathrin-coated vesicles: structural implications for coat assembly. J. Mol. Biol. *365*, 892–899.

Collins, B.M., McCoy, A.J., Kent, H.M., Evans, P.R., and Owen, D.J. (2002). Molecular architecture and functional model of the endocytic AP2 complex. Cell *109*, 523–535.

Conner, S.D., and Schmid, S.L. (2002). Identification of an adaptor-associated kinase, AAK1, as a regulator of clathrin-mediated endocytosis. J. Cell Biol. *156*, 921–929.

Doray, B., Lee, I., Knisely, J., Bu, G., and Kornfeld, S. (2007). The gamma/sigma1 and alpha/sigma2 hemicomplexes of clathrin adaptors AP-1 and AP-2 harbor the dileucine recognition site. Mol. Biol. Cell *18*, 1887–1896.

Fotin, A., Cheng, Y., Sliz, P., Grigorieff, N., Harrison, S.C., Kirchhausen, T., and Walz, T. (2004). Molecular model for a complete clathrin lattice from electron cryomicroscopy. Nature *432*, 573–579.

Gaidarov, I., Chen, Q., Falck, J.R., Reddy, K.K., and Keen, J.H. (1996). A functional phosphatidylinositol 3,4,5-trisphosphate/phosphoinositide binding domain in the clathrin adaptor AP-2 alpha subunit. Implications for the endocytic pathway. J. Biol. Chem. *271*, 20922–20929.

Heldwein, E.E., Macia, E., Wang, J., Yin, H.L., Kirchhausen, T., and Harrison, S.C. (2004). Crystal structure of the clathrin adaptor protein 1 core. Proc. Natl. Acad. Sci. USA *101*, 14108–14113.

Heuser, J.E., and Keen, J. (1988). Deep-etch visualization of proteins involved in clathrin assembly. J. Cell Biol. *107*, 877–886.

Höning, S., Ricotta, D., Krauss, M., Spate, K., Spolaore, B., Motley, A., Robinson, M., Robinson, C., Haucke, V., and Owen, D.J. (2005). Phosphatidylinositol-(4,5)-bisphosphate regulates sorting signal recognition by the clathrin-associated adaptor complex AP2. Mol. Cell *18*, 519–531.

Keen, J.H., Willingham, M.C., and Pastan, I. (1981). Clathrin and coated vesicle proteins Immunological characterization. J. Biol. Chem. *256*, 2538–2544.

Kelly, B.T., McCoy, A.J., Spate, K., Miller, S.E., Evans, P.R., Honing, S., and Owen, D.J. (2008). A structural explanation for the binding of endocytic dileucine motifs by the AP2 complex. Nature *456*, 976–979.

Kim, S.H., and Ryan, T.A. (2009). A distributed set of interactions controls mu2 functionality in the role of AP-2 as a sorting adaptor in synaptic vesicle endocytosis. J. Biol. Chem. *284*, 32803–32812.

Krissinel, E., and Henrick, K. (2007). Inference of macromolecular assemblies from crystalline state. J. Mol. Biol. *372*, 774–797.

Lee, I., Doray, B., Govero, J., and Kornfeld, S. (2008). Binding of cargo sorting signals to AP-1 enhances its association with ADP ribosylation factor 1-GTP. J. Cell Biol. *180*, 467–472.

Li, Y., Lu, W., Marzolo, M.P., and Bu, G. (2001). Differential functions of members of the low density lipoprotein receptor family suggested by their distinct endocytosis rates. J. Biol. Chem. *276*, 18000–18006.

Loerke, D., Mettlen, M., Yarar, D., Jaqaman, K., Jaqaman, H., Danuser, G., and Schmid, S.L. (2009). Cargo and dynamin regulate clathrin-coated pit maturation. PLoS Biol. *7*, e57.

Motley, A.M., Berg, N., Taylor, M.J., Sahlender, D.A., Hirst, J., Owen, D.J., and Robinson, M.S. (2006). Functional analysis of AP-2 alpha and mu2 subunits. Mol. Biol. Cell *17*, 5298–5308.

Ohno, H., Stewart, J., Fournier, M.C., Bosshart, H., Rhee, I., Miyatake, S., Saito, T., Gallusser, A., Kirchhausen, T., and Bonifacino, J.S. (1995). Interaction of tyrosine-based sorting signals with clathrin-associated proteins. Science *269*, 1872–1875.

Olusanya, O., Andrews, P.D., Swedlow, J.R., and Smythe, E. (2001). Phosphorylation of threonine 156 of the mu2 subunit of the AP2 complex is essential for endocytosis in vitro and in vivo. Curr. Biol. *11*, 896–900.

Owen, D.J., and Evans, P.R. (1998). A structural explanation for the recognition of tyrosine-based endocytotic signals. Science *282*, 1327–1332.

Owen, D.J., Collins, B.M., and Evans, P.R. (2004). Adaptors for clathrin coats: structure and function. Annu. Rev. Cell Dev. Biol. *20*, 153–191.

Paleotti, O., Macia, E., Luton, F., Klein, S., Partisani, M., Chardin, P., Kirchhausen, T., and Franco, M. (2005). The small G-protein Arf6GTP recruits the AP-2 adaptor complex to membranes. J. Biol. Chem. *280*, 21661–21666.

Pearse, B.M., and Robinson, M.S. (1984). Purification and properties of 100-kd proteins from coated vesicles and their reconstitution with clathrin. EMBO J. *3*, 1951–1957.

Ricotta, D., Conner, S.D., Schmid, S.L., von Figura, K., and Honing, S. (2002). Phosphorylation of the AP2 mu subunit by AAK1 mediates high affinity binding to membrane protein sorting signals. J. Cell Biol. *156*, 791–795.

Rohde, G., Wenzel, D., and Haucke, V. (2002). A phosphatidylinositol (4,5)-bisphosphate binding site within mu2-adaptin regulates clathrin-mediated endocytosis. J. Cell Biol. *158*, 209–214.

Rohrer, J., Schweizer, A., Russell, D., and Kornfeld, S. (1996). The targeting of Lamp1 to lysosomes is dependent on the spacing of its cytoplasmic tail tyrosine sorting motif relative to the membrane. J. Cell Biol. *132*, 565–576.

Saffarian, S., Cocucci, E., and Kirchhausen, T. (2009). Distinct dynamics of endocytic clathrin-coated pits and coated plaques. PLoS Biol. *7*, e1000191.

Schledzewski, K., Brinkmann, H., and Mendel, R.R. (1999). Phylogenetic analysis of components of the eukaryotic vesicle transport system reveals a common origin of adaptor protein complexes 1, 2, and 3 and the F subcomplex of the coatomer COPI. J. Mol. Evol. *48*, 770–778.

Schmid, E.M., and McMahon, H.T. (2007). Integrating molecular and network biology to decode endocytosis. Nature *448*, 883–888.

Semerdjieva, S., Shortt, B., Maxwell, E., Singh, S., Fonarev, P., Hansen, J., Schiavo, G., Grant, B.D., and Smythe, E. (2008). Coordinated regulation of AP2 uncoating from clathrin-coated vesicles by rab5 and hRME-6. J. Cell Biol. *183*, 499–511.

Spang, A., Matsuoka, K., Hamamoto, S., Schekman, R., and Orci, L. (1998). Coatomer, Arf1p, and nucleotide are required to bud coat protein complex I-coated vesicles from large synthetic liposomes. Proc. Natl. Acad. Sci. USA *95*, 11199–11204.

Traub, L.M. (2009). Tickets to ride: selecting cargo for clathrin-regulated internalization. Nat. Rev. Mol. Cell Biol. *10*, 583–596.

Voorhees, P., Deignan, E., van Donselaar, E., Humphrey, J., Marks, M.S., Peters, P.J., and Bonifacino, J.S. (1995). An acidic sequence within the cytoplasmic domain of furin functions as a determinant of trans-Golgi network localization and internalization from the cell surface. EMBO J. *14*, 4961–4975.

Zaremba, S., and Keen, J.H. (1983). Assembly polypeptides from coated vesicles mediate reassembly of unique clathrin coats. J. Cell Biol. *97*, 1339–1347.

## EXTENDED EXPERIMENTAL PROCEDURES

### Structure Determination of the AP2 Core in the Open Conformation

Recombinant AP2 cores were made in *E. coli* as in (Collins et al., 2002) from two bicistronic vectors. The first encoded α trunk-GST and untagged σ2 on a kanamycin resistance vector and His6-β2 trunk and myc tagged μ2 on an ampicillin resistance vector. Protein was expressed overnight at 22°C and affinity-purified with glutathione sepharose and NiNTA agarose, followed by gel filtration in 250 mM NaCl, 10 mM Tris pH 8 with 1 mM DTT. Crystals of the open form were grown from a mixture of AP2 cores (10 mg/ml) with the DYQRLN peptide derived from TGN38 (2 mg/ml) by hanging drop vapour diffusion against a reservoir containing 0.7 M lithium sulphate, 0.7 M ammonium sulphate and 200mM sodium citrate pH 7.4 with 5 mM DTT. Crystals were cryoprotected in mother liquor augmented with 20% glycerol and 3 mg/ml TGN38 peptide and all data were collected at 100K. Crystals were of space group R3 with unit cell dimensions a = 255 Å, c = 157 Å. Diffraction from all crystals was severely anisotropic, extending at best to around 3.1 Å resolution in the a-b plane, but not much beyond 5–6 Å resolution along c. The anisotropy of diffraction varied between crystals and many crystals were screened to maximise the resolution. Data were integrated with Mosflm (Leslie, 2006) and scaled with Scala (Evans, 2006). Heavy atom derivatives for multiple isomorphous replacement with anomalous scattering (MIRAS) experimental phasing were made using cryo-trapped Xe (10 atmospheres of pressure for 1 minute) and crystals soaked in the $Ta_6Br_{12}^{2+}$ cluster compound (see table). For the latter a single grain of $[Ta_6Br_{12}]Br_2$ was placed on the surface of a drop and left for 48 hr, during which time the crystals turned green, and then the crystals were transferred to cryoprotected mother liquor and flash frozen in liquid nitrogen. Data for the derivatives were collected using wavelengths giving a strong anomalous signal, for the $Ta_6Br_{12}^{2+}$ (“tantalum”) derivative, to 5 Å resolution, and for Xe to 3.5 Å resolution. The tantalum derivative was particularly sensitive to radiation damage, since the data were collected at the absorption edge. Six tantalum cluster sites were found with Hyss (Grosse-Kunstleve and Adams, 2003) and input to SHARP (Bricogne et al., 2003) for refinement and phasing. Log-likelihood gradient maps were used to find the Xe sites in the Xe derivative. The final phases were generated using native data to 3.1 Å resolution, a merged tantalum dataset from three crystals to 5 Å resolution, and a merged Xe dataset from three crystals to 3.5Å resolution. Phase information beyond 5 Å resolution, from the Xe derivative, was weak. Density modification by solvent flipping with Solomon (Abrahams, 1997) used 68% solvent. The high solvent content of the crystals is consistent with the low resolution of diffraction. A second Xe derivative dataset (Xe2 in Table S1) was added later, after the structure had been built and refined, to improve the experimental phases for refinement, particularly beyond 5 Å resolution.

The electron density clearly showed the α-helical solenoids for the α and β2 subunits and, less clearly, the location of the longin domains for σ2 and μ2. Even in the first experimentally phased electron density map, the dramatic nature of the conformational change was evident since there was no density corresponding to C-μ2 in its original position in the bowl in our two existing AP2 structures (Collins et al., 2002; Kelly et al., 2008), and there was weak density in an entirely different location. Since the conformation of β2 appeared to be largely conserved with the unlatched structure, and had good electron density, the β2 subunit was chosen as the first part to be docked into the electron density. This was achieved using phased molecular placement in MOLREP (Vagin and Isupov, 2001) (spherically averaged phased translation function + phased rotation function + phased translation function). Attempts were then made to dock fragments of the α subunit into the remaining good helical electron density in the experimentally phased map, but, unfortunately, the new fragments repeatedly docked erroneously into the electron density for β2. The remaining fragments were therefore placed using (unphased) molecular replacement with PHASER (McCoy et al., 2007), fixing the coordinates for β2 found by real-space docking. The α subunit was broken into three approximately equal fragments on the basis that the α subunit in the experimentally phased density appeared to be more bent than in the unlatched structure, and these three fragments, the σ2 and μ2 longin-like domains, and C-μ2 were used as separate models. The fragments of α and the σ2 and μ2 longin domains were found sequentially and unambiguously with high Z-scores and their position agreed with the experimental electron density. After docking the domains of the bowl it became apparent that β2 did not fit the experimental electron density very well at the N and C termini, and so several helices at the ends of the docked β2 domain were deleted and re-docked as separate rigid bodies. C-μ2 was more difficult to position into density due to its high B-factor in the crystal. The partial structure (without C-μ2) was refined in REFMAC (Murshudov et al., 1997) to improve the partial model and then molecular replacement with a loop-deleted model of C-μ2 was successful, fixing the refined partial structure (α, β2, N-μ2, σ2) coordinates. The positioning of C-μ2 agreed with the weak electron density for part of C-μ2 that could be seen in the original experimentally phased map. The structure was refined against the 3.1 Å resolution native data with phenix.refine (Adams et al., 2002) and REFMAC, using the experimental phases as restraints (MLHL target (Pannu et al., 1998)). Because of the severe anisotropy of the data and the comparatively poor quality of the maps, wherever possible the previous locked and unlatched structures of AP2 were used as guides for any necessary rebuilding of the model (using COOT (Emsley and Cowtan, 2004)), and in the later stages, restraints on main-chain hydrogen bonds in secondary structure elements were used, with helix and sheet definitions taken from the previous better-determined structures. The overall nature of the conformational changes from the locked structure are very clear from the electron density maps. Although the resolution was too poor for any detailed interpretation of individual side chains in most regions, in the best regions of the maps the pattern of large and small side chains matched that in the locked form, though de novo identification would have been impossible. Two notable new features were clear: firstly, part of the μ2 linker region that was disordered in the “locked” structure had now folded into a helix, packed in a groove between β2 and N-μ2. The new μ2 linker region was clearly helical, but in the absence of a template from the open

or unlatched forms the sequence registration had to be determined de novo, so a series of six models were constructed, covering the registrations consistent with loop distances: only one of these placed hydrophobic residues in positions buried in the β2/N-μ2 interface, with the hydrophilic residues exposed, so this one was chosen as the most likely model. Secondly, a long loop in C-μ2 that contains an inserted myc tag (MEQK**LI**, also disordered in the locked structure), wandered across a crystal contact into a neighbouring molecule and inserted into the dileucine-peptide binding site on σ2 (see main text and Figure 2C). This interaction forms the main crystal contact along the crystal c axis.

### Structure Determination of μ2-Truncated AP2 Core

Bicistronic plasmids expressing a form of the AP2 core that contained the same α,β2, and σ2 subunits as AP2 core except that only residues 1-150 of μ2 were present were constructed similarly to those used to express the full AP2 core (Collins et al., 2002). The protein was expressed and purified in the same manner as described above for the full length AP2 core, concentrated to 10 mg/ml and mixed with acidic dileucine peptide RMSEDEPLLSES. Crystals were grown by hanging drop vapour diffusion against a reservoir containing Hepes 0.1M pH 7 - 7.5, 5% MPD, EtOH 2%v:v. The crystals were of space group $P2_12_12_1$ and unit cell dimensions 174 Å x 176 Å x 192 Å, and were transferred to Hepes 0.1M pH 7 - 7.5, 7% MPD, EtOH 2% containing 22% glycerol and flash cooled to 100K. Data were collected at Diamond beamlines I02 and I03 and processed with Scala and Mosflm (Evans, 2006; Leslie, 2006). The crystals diffracted to a low resolution, and diffraction was also anisotropic. In the best direction the resolution extended to 4.5 Å but only extended to 6Å in the worst direction: the anisotropic delta-B was 196 Å$^2$. Although the data were poor, we were able to determine which of the three models for the AP2 bowl (comprising α, β2, N-μ2, and σ2) was most consistent with the data. Molecular replacement using PHASER (McCoy et al., 2007) was attempted with models derived from the open, unlatched and locked structures by deleting the C-μ2 domain. Only the open form gave a solution with high Z-scores. There were three molecules in the asymmetric unit and a solvent content of 50%. Searches with the "unlatched" and locked formations failed to find solutions with high Z-scores that packed more than one molecule in the asymmetric unit (and formed a lattice).

***μ2-Truncated Core Crystallographic Data***

| | Crystal Form P1 | Crystal Form $P2_12_12_1$ |
|---|---|---|
| Beamline | Diamond I02 & I03 | Diamond I02 & I03 |
| Wavelength (Å) | 0.92 | 0.92 |
| Resolution (Å) | 5.0 | 6.0 |
| $R_{merge}$ | 0.262 | 0.230 |
| $R_{meas}$ | 0.371 | 0.250 |
| Completeness | 88.8% | 99.7% |
| Multiplicity | 1.8 | 6.8 |
| <<I/>σ<I>> | 1.9 | 4.7 |
| Wilson plot B(Å$^2$) | 450.0 | 220.2 |
| Solvent content (%) | 59.2 | 50.0 |

***Molecular Replacement Attempts of $P2_12_12_1$ Form of μ2-Truncated Core***

| | *RFZ* | *TFZ* | *PAK* | *LLG* |
|---|---|---|---|---|
| *Using Open Form as Search Model* | | | | |
| First | 4.6 | 15.3 | 12 | 160 |
| Second | 2.7 | 25.1 | 18 | 505 |
| Third | 2.7 | 7.2 | 23 | 583 |
| *Using Unlatched Form as Search Model* | | | | |
| First | 3.9 | 4.8 | 0 | 53 |
| Second | 4.0 | 8.3 | 787 | — |
| *Using Locked Form as Search Model* | | | | |
| First | 3.5 | 4.7 | 0 | 40 |
| Second | 4.4 | 8.5 | 718 | — |

A second crystal form of space group P1 and unit cell dimensions 159 Å x 160 Å x 178 Å, 90.0°, 90.0°, 111.2° grew in condition of Hepes 0.1M pH 7 - 7.5, PEG 4k 10-13%, 0.1 M Na acetate using the same protein:peptide mix. Data were collected as above. The crystals diffracted to slightly higher resolution than the orthorhombic crystal form and diffraction was less anisotropic. In the best direction the resolution extended to 4.5 Å and to 5 Å in the worst direction: the anisotropic delta-B was 78 Å$^2$. Again, molecular replacement trials with PHASER (McCoy et al., 2007) with the open, unlatched and locked bowl structures showed that the open

structure was most consistent with the data since no solutions could be found using either the unlatched or locked conformations of the bowl as search models. There were seven molecules in the asymmetric unit and a solvent content of 59%.

**Molecular Replacement Attempts of P1 Form of μ2-Truncated Core**

| | *RFZ* | *TFZ* | *PAK* | *LLG* |
|---|---|---|---|---|
| First | 3.6 | 100 | 0 | 87 |
| Second | 4.4 | 7.2 | 0 | 204 |
| Third | 3.2 | 12.5 | 0 | 369 |
| Fourth | 3.2 | 8.6 | 1 | 491 |
| Fifth | 3.2 | 11.0 | 5 | 663 |
| Sixth | 2.4 | 13.9 | 10 | 833 |
| Seventh | 2.9 | 15.8 | 21 | 1060 |

We consider the resolution of these crystal forms too low for all-atom refinement or detailed molecular analysis: however these data indicate that the conformation of the bowl seen in the truncated and open, motif-liganded forms of the AP2 core are the same, and that this serves to validate the "open" conformation as the stable, low energy conformational state of the α–β2 bowl.

## Liposome-Based SPR Studies

### *Peptides Sequences and Conjugation of Peptides to Lipid*

Sorting motif-containing peptides derived from the cytoplasmic tails of TGN 38 (CKVTRRPKASDYQRL) and phosphorylated CD4 (CHRRRQAERM(S*P)*QIKRLLSEK) were synthesized with an amino-terminal cysteine. The quality of all peptides was controlled by mass spectrometry after reverse-phase chromatography. For covalent conjugation of a peptide to a synthetic lipid, 5 mmol peptide in 1 ml 10 mM MOPS-KOH pH 7.5, 50% DMF were mixed with an equal volume of lipid (1,2-Dipalmitoyl-sn-Glycero-3-Phosphoethanolamine-N-[4-(p-maleimidophenyl)butyramide], Avanti, USA) at 5 mg/ml in Chloroform and incubated for 2 hr at 20°C with end-over-end rotation. Coupling was blocked by addition of ß-Mercaptoethanol to a final concentration of 10mM and further incubation for 30 min. The lipid-linked peptide was collected from the organic phase after extraction with 2 ml Chloroform and 1ml Methanol and dried under Nitrogen. The dried peptido-lipid was resuspended in Chloroform/Methanol (2:1) at 5 mg/ml and stored at −20°C.

## Synthesis of Liposomes

All lipids (phosphatidylcholine, phosphatidylethanolamine, and the phosphoinositide $PtdIns4,5P_2$) were stored in chloroform/methanol in a nitrogen atmosphere. For preparation of basic liposomes (80% PC, 20% PE), the appropriate amounts (w/w) were mixed and dried under nitrogen and rehydrated with 150 μl of 0.3M sucrose for 1 hr before the volume was adjusted to 1ml using $H_2O$. The liposomes were then sedimented by centrifugation at 20,000 rpm for 1 hr at 4°C (TLA 120 rotor, Beckman tabletop) and resuspended in 500 μl 20 mM HEPES, pH 7.4. To prepare unilamellar liposomes the liposome solution was passed 15-times through an extruder using a 100 nm membrane (LiposoFast, Avestin, Canada) and kept for further use at 4°C. Complex liposomes were prepared by substitution of 10% PC by the equivalent amount of lipid-linked peptide and/or one of the mentioned phosphoinositides.

## Biosensor Experiments

Peptido-liposomes were used to generate a stable "membrane mimic" on an L1 sensor surface of a BIAcore 3000 (BIAcore AB, Sweden) SPR biosensor. The system was first equilibrated in 10 mM Tris pH 8.7, 250 mM NaCl 1 mM DTT, which was used as the running buffer followed by priming with two injections of 20 mM CHAPS for 1 min (flow rate 10 μl/min) and injection of the liposomes (0.25 mM final concentration) for 4 min at 5 μl/min. Loosely bound liposomes were removed by two pulse injections of 50 mM sodium hydroxide for 30 s at 30 μl/min. This procedure resulted in an increase of the baseline by app. 9500 RU with less than 4% variation between the four flow-cells. After liposome capture, wild-type and mutant AP2 cores were injected at concentrations ranging from 50 nM to 1 μM at a flow rate of 30 μl/min for one min (association) followed by buffer flow for 4 min (dissociation). All protein that did not dissociate within this period from the membrane was stripped off by a 20 s pulse injection of 50 mM NaOH.

All AP2 complexes used in the SPR binding assay expressed to similar levels and were checked for intactness and folding by SDS-PAGE and circular dichrosim prior to use (data not shown). The kinetic analysis of binding was carried out as described (Ricotta et al., 2002) and rate constants were calculated using the Evaluation software supplied by the manufacturer. SPR experiments were repeated 3 to 5 times and included the calculation of the kinetic rate constants. The $K_D$ values presented represent the mean values with a variance lower than 8%.

## Fluorescence Anisotropy Measurements (Equilibrium Binding)

A peptide encoding the TGN38 YxxΦ motif (sequence ASDYQRL) and modified at its N-terminus with fluorescein (Sigma-Genosys) was used in all binding experiments at a concentration of 20 nM. Various quantities of recombinant AP2 core or isolated C-μ2 (prepared as in (Owen and Evans, 1998)) were premixed with the fluorescent peptide and incubated for ∼10 minutes to allow the mixture to come to equilibrium. Protein / peptide mixtures were prepared in 10 mM Tris pH 8.7, 250 mM NaCl (for AP2 core / short

core) or 10 mM HEPES pH 7.4, 500 mM NaCl (for C-μ2). Fluorescence anisotropy measurements were carried out at 21°C with a PheraStar Plus plate reader (BMG Labtech) using a 485/520 nm fluorescence polarisation module. With these excitation and emission wavelengths there was no significant change in total fluorescence of the peptide when binding was saturated with C-μ2 (data not shown). Where used, polymeric heparin (~50 subunits) (Rovi Laboratories) or heparin disaccharide (Iduron) was added to 500 μM concentration. Mean fluorescence anisotropy values (3 or more measurements) were plotted against protein concentration and the curves fitted to a single-site binding model:

$$F = F_f + (F_b - F_f).[L]/(K_d + [L]) + C_{ns}.[L]$$

where F is the measured anisotropy in a mixture of free (f) and bound (b) fluorescent peptide with anisotropy of $F_f$ and $F_b$ respectively, [L] is the pseudo-first order concentration of "ligand" (in this case, C-μ2 or AP2), $K_d$ is the equilibrium dissociation constant and $C_{ns}$.[L] (where $C_{ns}$ is a constant) represents the linear contribution from nonspecific binding. Curves were fitted with ProFit software using the Levenberg-Marquardt algorithm. Figure S5 shows data plots and curve fits.

In addition to AP2 core and isolated C-μ2, binding titrations were performed with μ2-truncated core as a negative control (Figure S5). Since this construct lacks the C-μ2 subdomain, it is unable to bind the YxxΦ motif and thus any observed binding is due to nonspecific interactions. The binding curves for μ2-truncated core (both with and without the addition of heparin) and AP2 core (without heparin) do not differ significantly and both can be fitted to straight lines (R = 0.99); thus, no specific binding is observed for AP2 in the absence of heparin in this equilibrium assay. The addition of ~50-mer heparin had no effect on the association of short core with the peptide, whereas binding of AP2 core was stimulated. The addition of heparin disaccharide to AP2 core did not stimulate binding.

### Stopped-Flow Fluorescence Anisotropy (Pre-Steady-State Kinetics)

The same fluorescent peptide used for equilibrium studies was used throughout. Relaxation times were determined for a range of μM C-μ2 and AP2 concentrations at pseudo-first order excess and used to determine kinetic rate constants. The anisotropy change was fitted to a single exponential function:

$$F = \Delta F \exp(-k_{app} t) + F_e,$$

where $F$ is the observed fluorescence anisotropy, $\Delta F$ is the fluorescence anisotropy amplitude, $k_{app}$ is the apparent pseudo first-order relaxation rate, which is equivalent to the inverse relaxation time (1/t), and $F_e$ is the end-point fluorescence anisotropy.

The on-rate ($k_{on}$) and off-rate ($k_{off}$), and thus the dissociation constant (= $k_{off}/k_{on}$) were derived by fitting the apparent pseudo first-order relaxation rates to the following equation:

$$k_{app} = k_{off} + k_{on}.C$$

where C is the concentration of C-μ2 or AP2, as appropriate.

## SUPPLEMENTAL REFERENCES


Abrahams, J.P. (1997). Bias reduction in phase refinement by modified interference functions: introducing the γ function. Acta Crystallogr. D Biol. Crystallogr. *53*, 371–376.

Adams, P.D., Grosse-Kunstleve, R.W., Hung, L.W., Ioerger, T.R., McCoy, A.J., Moriarty, N.W., Read, R.J., Sacchettini, J.C., Sauter, N.K., and Terwilliger, T.C. (2002). PHENIX: building new software for automated crystallographic structure determination. Acta Crystallogr. D Biol. Crystallogr. *58*, 1948–1954.

Bricogne, G., Vonrhein, C., Flensburg, C., Schiltz, M., and Paciorek, W. (2003). Generation, representation and flow of phase information in structure determination: recent developments in and around SHARP 2.0. Acta Crystallogr. D Biol. Crystallogr. *59*, 2023–2030.

Collins, B.M., McCoy, A.J., Kent, H.M., Evans, P.R., and Owen, D.J. (2002). Molecular architecture and functional model of the endocytic AP2 complex. Cell *109*, 523–535.

Emsley, P., and Cowtan, K. (2004). Coot: Model-building tools for molecular graphics. Acta Crystallogr. D Biol. Crystallogr. *60*, 2126–2132.

Evans, P. (2006). Scaling and assessment of data quality. Acta Crystallogr. D Biol. Crystallogr. *62*, 72–82.

Grosse-Kunstleve, R.W., and Adams, P.D. (2003). Substructure search procedures for macromolecular structures. Acta Crystallogr. D Biol. Crystallogr. *59*, 1966–1973.

Heller, H., Schaefer, M., and Schulten, K. (1993). Molecular-dynamics simulation of a bilayer of 200 lipids in the gel and in the liquid-crystal phases. J. Phys. Chem. *97*, 8343–8360.

Kelly, B.T., McCoy, A.J., Spate, K., Miller, S.E., Evans, P.R., Honing, S., and Owen, D.J. (2008). A structural explanation for the binding of endocytic dileucine motifs by the AP2 complex. Nature *456*, 976–979.

Krissinel, E., and Henrick, K. (2005). Detection of Protein Assemblies in Crystals (Berlin: Springer-Verlag).

Leslie, A.G. (2006). The integration of macromolecular diffraction data. Acta Crystallogr. D Biol. Crystallogr. *62*, 48–57.

McCoy, A.J., Grosse-Kunstleve, R.W., Storoni, L.C., Adams, P.D., and Read, R.J. (2007). PHASER crystallographic software. J. Appl. Cryst. *40*, 658–674.

Murshudov, G.N., Vagin, A.A., and Dodson, E.J. (1997). Refinement of macromolecular structures by the maximum-likelihood method. Acta Crystallogr. D Biol. Crystallogr. *53*, 240–255.

Owen, D.J., and Evans, P.R. (1998). A structural explanation for the recognition of tyrosine-based endocytotic signals. Science *282*, 1327–1332.

Owen, D.J., Vallis, Y., Noble, M.E., Hunter, J.B., Dafforn, T.R., Evans, P.R., and McMahon, H.T. (1999). A structural explanation for the binding of multiple ligands by the alpha-adaptin appendage domain. Cell *97*, 805–815.

Owen, D.J., Vallis, Y., Pearse, B.M., McMahon, H.T., and Evans, P.R. (2000). The structure and function of the beta 2-adaptin appendage domain. EMBO J. *19*, 4216–4227.

Pannu, N.S., Murshudov, G.N., Dodson, E.J., and Read, R.J. (1998). Incorporation of prior phase information strengthens maximum-likelihood structure refinement. Acta Crystallogr. D Biol. Crystallogr. *54*, 1285–1294.

Potterton, L., McNicholas, S., Krissinel, E., Gruber, J., Cowtan, K., Emsley, P., Murshudov, G.N., Cohen, S., Perrakis, A., and Noble, M.E. (2004). Developments in the CCP4 molecular-graphics project. Acta Cryst. *D60*, 2288–2294.

Ricotta, D., Conner, S.D., Schmid, S.L., von Figura, K., and Honing, S. (2002). Phosphorylation of the AP2 mu subunit by AAK1 mediates high affinity binding to membrane protein sorting signals. J. Cell Biol. *156*, 791–795.

Schneider, T.R. (2002). A genetic algorithm for the identification of conformationally invariant regions in protein molecules. Acta Crystallogr. D Biol. Crystallogr. *58*, 195–208.

Vagin, A.A., and Isupov, M.N. (2001). Spherically averaged phased translation function and its application to the search for molecules and fragments in electron-density maps. Acta Crystallogr. D Biol. Crystallogr. *57*, 1451–1456.

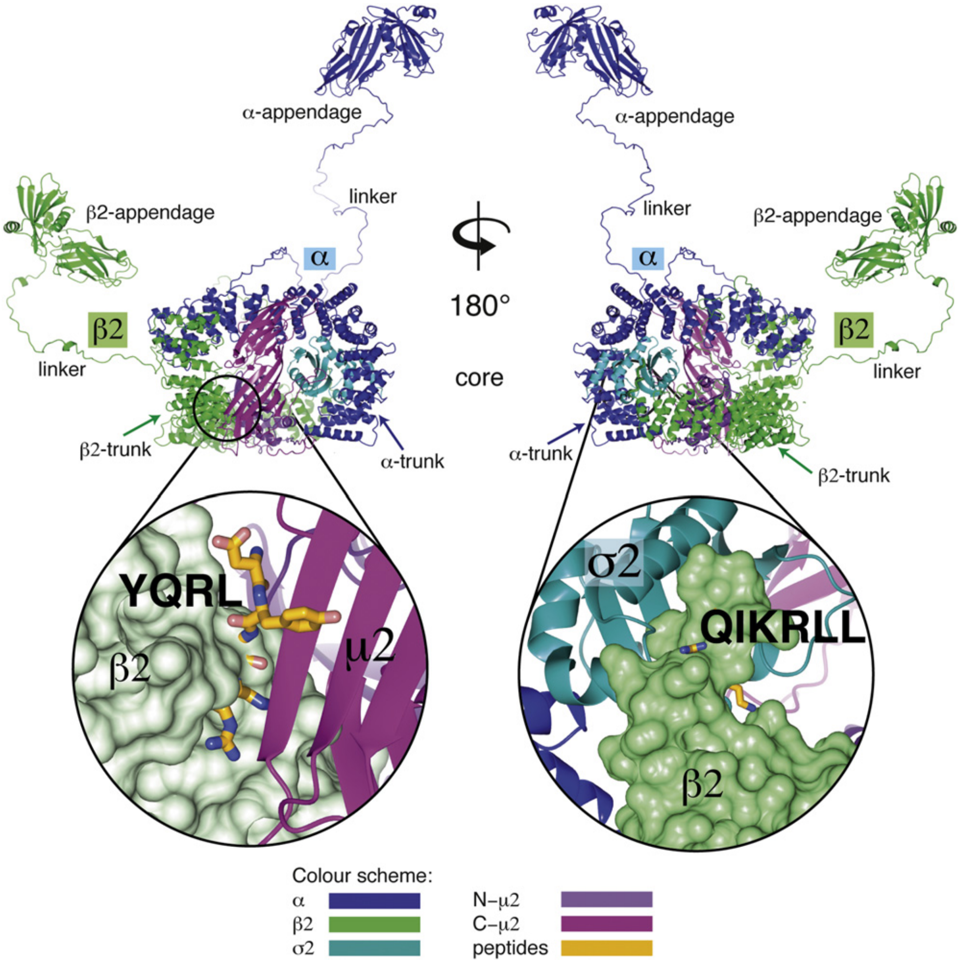


**Figure S1. The Cargo Motif-Binding Sites of AP2 Are Blocked in the Locked Structure, Related to Figure 1**

Top: overall composite view of the whole AP2 complex in its locked conformation. The "core" consists of the smaller σ2 and μ2 subunits, along with the N-terminal "trunk" domains of the long α and β2 subunits (Collins et al., 2002). These are linked to the folded appendage domains (Owen et al., 1999; Owen et al., 2000) by unstructured linker regions. Bottom: close-up of the two blocked sites for cargo motifs. Left: the YxxΦ site (gold peptide YQRL) on C−μ2 (magenta) is blocked by β2 (green surface). Right: the acidic dileucine site (yellow peptide QIKRLL) on σ2 (cyan) is blocked by the N-terminus of β2, with β2Tyr6 and β2Phe7 in the place of LL. All molecular figures throughout were made with CCP4MG (Potterton et al., 2004).

Cell

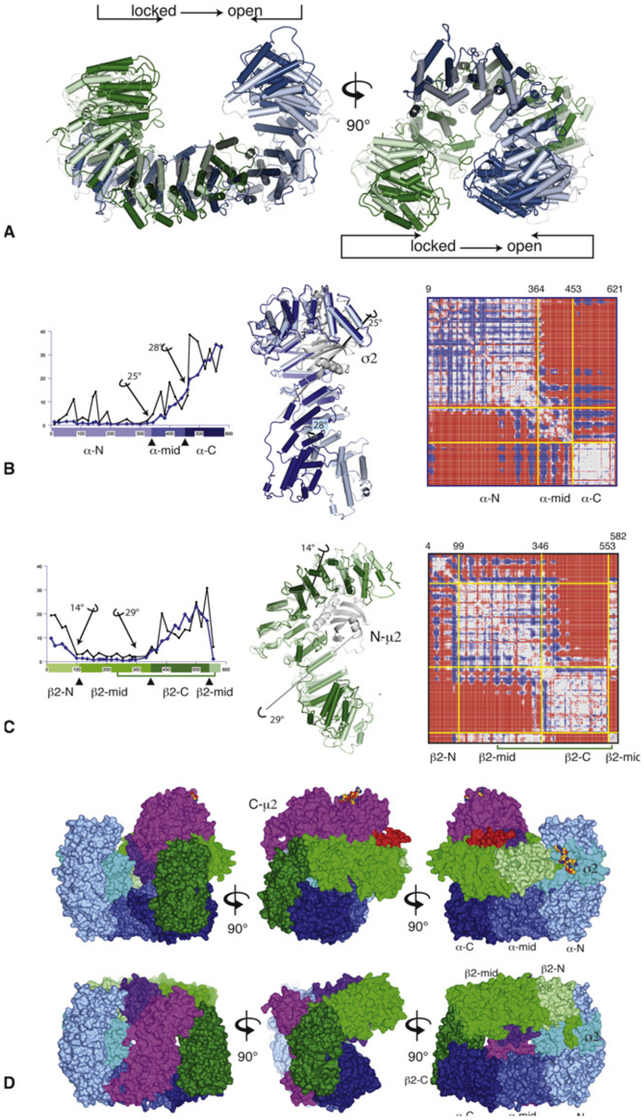


**Figure S2. Conformational Changes in the α and β2 Subunits, Related to Figure 3**

(A) In going from the locked conformation (pale colours, α blue, β2 green) to the open conformation (dark colors), the α/β2 solenoid bowl collapses inwards into the space vacated by C-μ2, as shown by the arrows. See also Movie S1 and Movie S2.

(B) Analysis of the conformational change in the α subunit. The open and locked structures were superimposed on the σ2 subunit (white). The graph shows the shift (blue line) and relative rotation angle (black line) of each helix along the sequence. The conformational change can be approximated as hinge rotations of 25° and 28° between three rigid bodies (α-N, α-mid and α-C, light to dark blue, hinge points at residues 342 and 460 marked with triangles). In the centre, the open conformation is in dark blue, and the locked conformation in light blue, with the hinge axes indicated. The right-hand panel is a difference distance plot (Schneider, 2002) coloured from red (negative differences) through white (small differences) to blue (positive differences), showing the division into three mainly rigid groups.

(C) Analysis of the conformational change in the β2 subunit. As in (A), except the structures were superimposed on the N-μ2 domain (white). The β2 subunit falls roughly into three rigid groups with hinge points shown at residues 98 and 346, coloured light to dark green, β2-N, β2-mid and β2-C. The C-terminal region folds back to join the middle region from residue 553, and the extreme N-terminus (residues 4-12) has an unrelated conformation. Centre and right panels, ribbon drawing and difference distance plot, as in (B).

(D) Rigid groups in the conformational change. Three orthogonal views (left to right) of the open (top) and locked (bottom) conformations, coloured by the rigid groups. The rigid groups in α and β2 are coloured light to dark blue and green respectively. The C-terminal regions of the α and β2 solenoids (α-C and β2-C) form a single rigid group. Other colours: σ2 cyan; N-μ2 purple; C- μ2 magenta; μ2 linker (142-160), red.

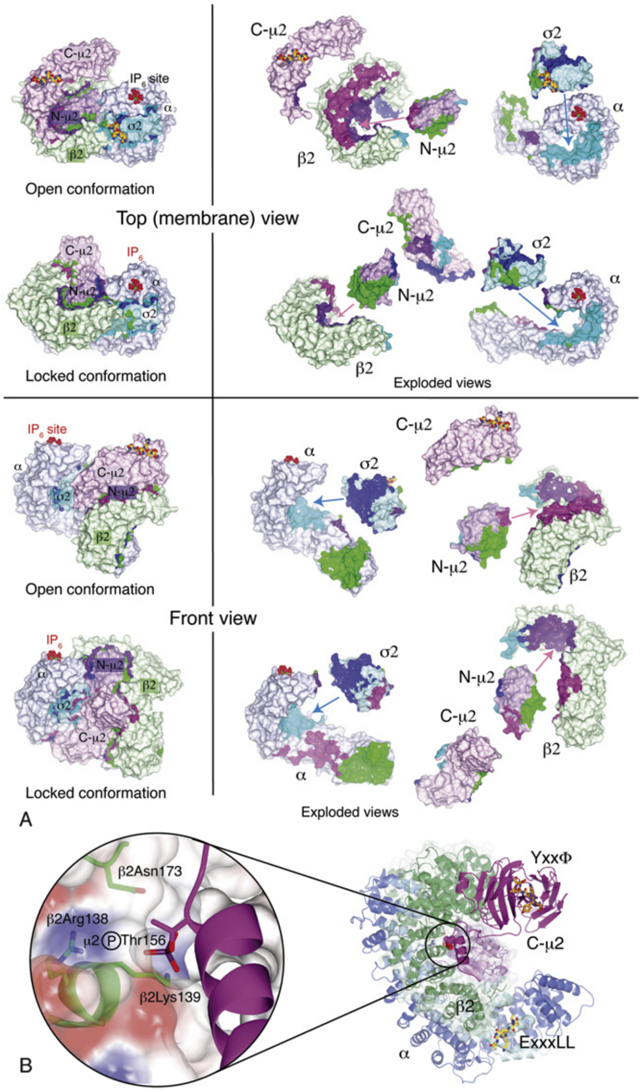


**Figure S3. Subunit Contacts in the AP2 Core, Related to Figure 4**

(A) Two views of the open and locked conformations are shown, as a whole and in "exploded" views. The interacting surface patches of each subunit are coloured according to the subunit that they contact: α, blue; β2 green; σ2 cyan; Nμ-2 purple; C-μ2 magenta. Interacting surfaces were calculated and analysed in CCP4MG (Potterton et al 2004).

(B) Left: a model of a phosphoryl group attached to the side chain identified as μ2Thr156, showing that it can fit in a positively charged pocket created by β2Arg138 and β2Lys139. β2Asn173 seems to form a hydrogen bond to μ2Thr156 in the structure. No attempt was made to model possible rearrangements around the site, apart from the threonine side chain. Right: structure of the whole AP2 core identifying the position of the μ2 linker and its potential phosphorylation site, which is shown enlarged on the left.

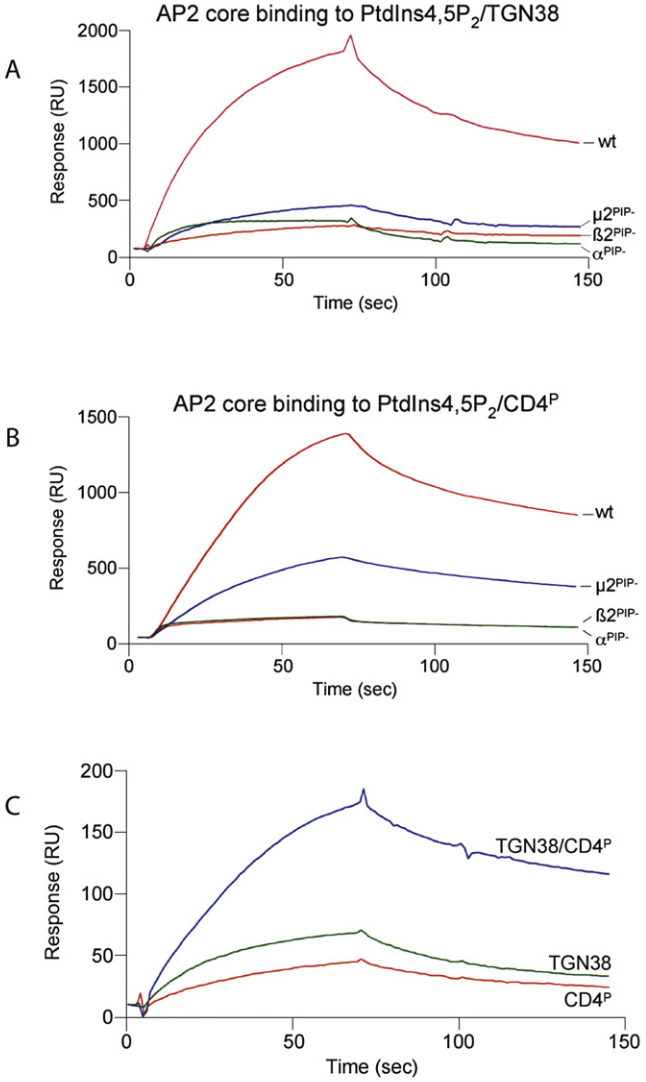


**Figure S4. Binding of AP2 Core to Liposomes of Various Phospholipid and Cargo Peptide Composition, Related to Figure 5**

(A and B) Wild type AP2 Core and mutants affecting the PtdIns4,5$P_2$ binding sites in $\alpha$, $\beta$2 and $\mu$2 (all at 200 nM) were passed over sensor surfaces with the Yxx$\Phi$ sorting motif from TGN38 (A) and the dileucine sorting signal of CD4 (B), both embedded in PtdIns4,5$P_2$-containing liposomes. The displayed sensorgrams are corrected for non-specific binding to PC/PE liposomes. The rate constants for all interactions are given in the Table in Figure 5C and were calculated from experiments in which AP2 core binding was recorded for five different concentrations ranging from 100 nM to 20 $\mu$M.

(C) Binding of AP2 to PtdIns4,5$P_2$ liposomes containing either or both Yxx$\Phi$ and acidic dileucine sorting motifs. The four flow cells of a L1 sensor surface were derivatized with liposomes containing 10% PtdIns4,5$P_2$ (flow cell 1), 10% PtdIns4,5$P_2$ + 1% Yxx$\Phi$ signal (flow cell 2), 10% PtdIns4,5$P_2$ + 1% dileucine signal (flow cell 3) or 10% PtdIns4,5$P_2$ + 0.5% of each signal (flow cell 4). After a pulse injection with NaOH to stabilize the surface, the binding of the AP2 core was recorded as above. The sensorgram shows binding of 200 nM AP2 core to the indicated liposomes after subtraction of the binding level obtained for PtdIns4,5$P_2$ liposomes.

Cell

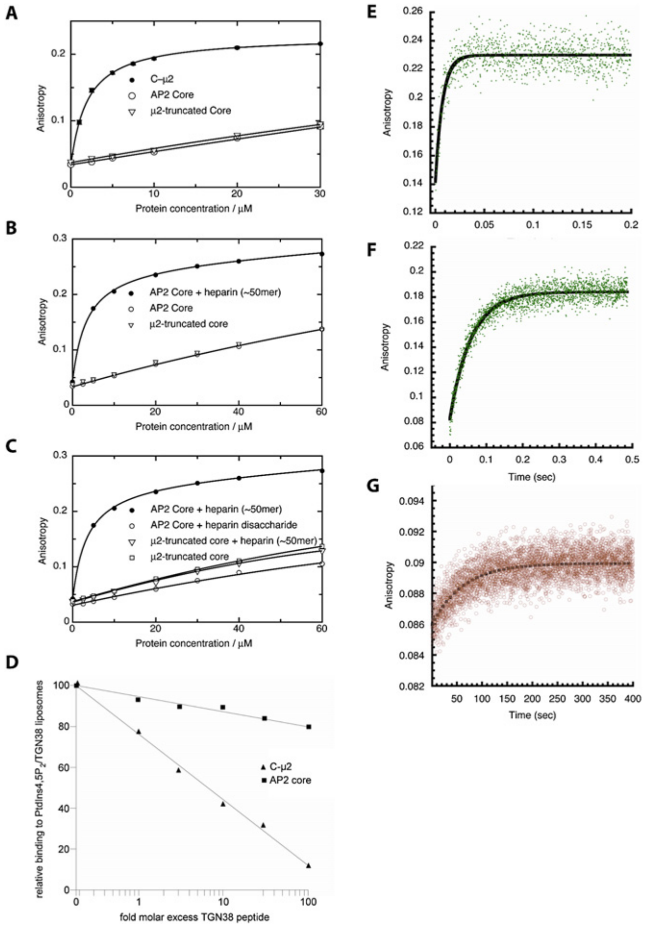


**Figure S5. Related to Figure 6**

(A–C) Fluorescence anisotropy equilibrium binding curves. Fluorescence anisotropy was measured for varying pseudo-first order concentrations of protein (as indicated) mixed with fluorescent YxxΦ peptide as described in Experimental Procedures. (A) Comparison of C-μ2 with AP2; (B) comparison of AP2 with and without the addition of heparin; and (C) the effect of heparin chain length. Means of three or more measurements are plotted. Standard error is omitted for clarity but typically was in the range 0.001-0.004.

(D) Competition of Core binding to YxxΦ/PtdIns4,5$P_2$ liposomes by free YxxΦ peptide. 200 nM of either AP2 core or C-μ2 were incubated with the indicated concentrations of the TGN38 YxxΦ-containing peptide for 15 min at room temperature. Subsequently, protein with peptide was passed over PtdIns4,5$P_2$/TGN38-containing liposomes. Binding was recorded for 2 min. The maximum binding levels for protein without addition of peptide were set to 100%.

(E–G) Relaxation rates for Yxxφ-peptide interaction with C-μ2 and AP2 in the presence or absence of heparin. Protein and peptide were rapidly mixed in a stopped-flow spectrometer and the change in anisotropy that occurs upon binding was measured. The anisotropy change was fit to a single exponential to obtain relaxation times. Yxxφ binds to 15μM C-μ2 with a relaxation time (t) = 0.0073 s (E), to 15μM AP2 in the presence of heparin with t = 0.059 s (F) and to 30μM AP2 in the absence of heparin with t = 62.5 s (G).
